# Effect of alcohol on clinical complications of hepatitis virus-induced liver cirrhosis: a consecutive ten-year study

**DOI:** 10.1186/s12876-022-02198-w

**Published:** 2022-03-19

**Authors:** Kodjo-Kunale Abassa, Xiao-Ying Wu, Xiu-Ping Xiao, Hao-Xiong Zhou, Yun-Wei Guo, Bin Wu

**Affiliations:** 1 Department of Gastroenterology, The Third Affiliated Hospital of Sun Yat-Sen University, 600 Tianhe Road, Guangzhou, 510630 China; 2 Guangdong Provincial Key Laboratory of Liver Disease Research, Guangzhou, China; 3 Department of Clinical Laboratory, The Third Affiliated Hospital of Sun Yat-Sen University, Guangzhou, China

**Keywords:** Ethanol, Hepatitis B virus, Hepatitis C virus, Liver cirrhosis, Complications

## Abstract

**Background and aims:**

Although coexisting alcohol-induced liver disease and hepatitis B or C virus-induced liver cirrhosis (ALD + HBV or ALD + HCV) has been the center of recent hepatology researches, numerous controversies still persist. This study aimed to showcase the influence of alcohol on the laboratory values and on the clinical outcomes of patients with hepatitis B and C virus-induced liver cirrhosis.

**Methods:**

Patients diagnosed with liver cirrhosis (n = 22,287) from January 2010 to December 2019 were enrolled, and divided into five groups according to the etiology: alcohol-induced liver disease (ALD, 1652 cases), hepatitis B virus (HBV, 18,079 cases), hepatitis C virus (HCV, 682 cases), ALD + HBV (1594 cases) and ALD + HCV (280 cases). Laboratory results and proportion of different liver cirrhosis complications were contrasted between groups.

**Results:**

The proportions of patients with Child Pugh grade C (28.0% vs 18.8%, *P* < 0.001) or MELD greater than 18 (24.1% vs 18.5%, *P* < 0.001) in the ALD + HBV group exceeded significantly those in the HBV group. Multivariate logistic regression revealed that the risk of hepatocellular carcinoma (HCC) and that of esophageal gastric variceal bleeding (EGVB) in the ALD + HBV group was respectively 2.01-fold and 1.74-fold that in the HBV group (HCC: OR = 2.01, 95% CI [1.58–2.55]; EGVB: OR = 1.74, 95% CI [1.30–2.33]) after adjusting for potential confounders. Furthermore, a linear-by-linear analysis test showed a decrease in the risk of HCC and EGVB with the duration of alcohol abstinence. Moreover, patients with both antiviral treatment and alcohol abstinence had the lowest risk of HCC and EGVB (HCC: OR = 0.10, 95% CI [0.05–0.20], *P* < 0.001; EGVB: OR = 0.17, 95% CI [0.06–0.45], *P* < 0.001) compared to those without any treatment, those with abstinence alone and those with antiviral therapy alone. Similar pattern was noticed while comparing the ALD + HCV group to the HCV group.

**Conclusion:**

Heavy alcohol use increased the severity of liver function impairment and the prevalence of HCC and EGVB in hepatitis virus-induced liver cirrhosis patients. Remarkably, long-term alcohol abstinence coupled with antiviral treatment effectively decreased the risk of HCC and EGVB in these populations.

**Supplementary Information:**

The online version contains supplementary material available at 10.1186/s12876-022-02198-w.

## Introduction

Alcohol use disorder, HBV and HCV are the three major causes of cirrhosis worldwide and in China. Yearly mortality associated with HCV-related diseases including cirrhosis and HCC is estimated to approximately 700,000 worldwide [1]. By date, over 350 millions people are estimated to be chronically infected by HBV worldwide [2]. Due to the considerable progress achieved by vaccination programs and treatment of hepatitis B and C, the prevalence of these two entities has significantly decreased over the decades. However, the actual socioeconomic development of the Chinese population participated in the rise of the prevalence of alcohol-related liver disease (ALD) in China [3], leading to an increase in cases of coexisting ALD and hepatitis virus B or C patients (ALD + HBV, ALD + HCV). According to WHO, over 3 million people die yearly due to heavy alcohol use. This represents approximately 6% of the overall deaths worldwide. ALD is a spectrum of disease ranging from steatosis to steatohepatitis, fibrosis and even hepatocarcinoma. The conventionally used biomarkers for the diagnosis of ALD include elevated MCV and GGT, AST/ALT greater than 1.5, etc. Even though the specificity and sensitivity of these markers are limited, they still play an important role in the suspicion and diagnosis of ALD [4]. However, some controversies still exist on the extent to which the presence of ALD together with viral hepatitis affects these laboratory markers.

The interaction between alcohol and hepatitis virus B or C has been widely studied recently with non-unanimous conclusions [5]. Heavy alcohol consumption had been shown to accelerate the development of liver fibrosis, and to increase the prevalence of HCC and mortality in HCV infected patients [6–8]. Concerning the interaction between alcohol and HBV, less conclusive studies has been done. Some studies showed an increase risk of HCC in HBV infected patients with heavy alcohol consumption habit, whereas other estimates that small to moderate alcohol use did not increase the risk of fibrosis in HBV patients [9]. Nevertheless, more need to be explored about the prevalence of other liver cirrhosis complications such as EGVB among these groups of patients. Therefore, this studied has been designed to assess the effect of alcohol in HBV and HCV cirrhotic patients by, first comparing disease severity between ALD, HBV and HCV patients and also between ALD + HBV and HBV, and between ALD + HCV and HCV, using commonly available biomarkers and disease severity assessment scores; then evaluate the prevalence of different liver cirrhosis complications between these groups.

## Materials and methods

### Patients enrollment and data acquisition

Medical records of all inpatient subjects diagnosed for the first time with liver cirrhosis in the third affiliated hospital of Sun Yat-Sen University, Guangzhou, China from January 2010 to December 2019 were retrospectively collected from the hospital electronic database of medical record. The identification of the cirrhosis cases was made using ICD-10-CM codes of liver cirrhosis (K74.100). Patients with Wilson Disease, autoimmune liver disease, congenital biliary atresia, multiple etiologies other than ALD + HBV and ALD + HCV, unknown etiology, and those with incomplete data were excluded. The remaining cases were divided into five groups based on the etiology of cirrhosis: alcohol-induced liver cirrhosis (ALD group), hepatitis B virus-induced liver cirrhosis (HBV group), hepatitis C virus-induced liver cirrhosis (HCV group), coexisting ALD and HBV (ALD + HBV), and coexisting ALD and HCV (ALD + HCV). Information such as sex, age at admission, laboratory data, clinical complications and history of antiviral treatment, alcohol consumption and alcohol abstinence were collected. Different liver function assessment scores such as Child–Pugh classification, MELD (model for end-stage liver disease) score, GAHS (Glasgow alcoholic hepatitis score) and MDS (Maddrey’s discriminant function Scale) were calculated. The calculation formulas and criteria for diagnosis of each type of cirrhosis and its complications is detailed in the Additional file 1.

### Patients’ stratification and statistical analysis

We first compared the severity of liver function impairment between the groups using laboratory data such as ALT, AST, Child classification, MELD score, GAHS and MDS. The proportion of different liver cirrhosis complications such as HCC, infections, ascites, EGV, EGVB, hepatic encephalopathy (HE) and hepatorenal syndrome (HRS), was also evaluated among the groups. Multivariate logistic regressions were conducted to evaluate the association between different influencing factors such as etiology and the risk of HCC and EGVB. We then assessed the difference in the proportion of HCC and EGVB between males and females in each etiology group. Next, patients in the ALD, ALD + HBV and ALD + HCV groups were stratified into four subgroups according to the duration of their abstinence from alcohol: no abstinence, abstinent less than five years, abstinent five to ten years and abstinent more than ten years. Abstinence from alcohol was defined as restraint from any alcohol beverage for more than six months. Patients with unclear history of alcohol abstinence were excluded from this part of the study. The relation between alcohol abstinence and risk of HCC and EGVB was assessed in different groups through linear-by-linear association test and logistic regression analysis. Finally, to evaluate how antiviral treatment (AVT) affects the frequency of HCC and EGVB in the studied population, we divided HBV and HCV patients into two subgroups (patients with AVT, and those without AVT). The ALD + HBV patients and ALD + HCV patients were divided into 4 subgroups (patients without AVT and no alcohol abstinence, patients without AVT with alcohol abstinence, patients with AVT without alcohol abstinent and patients with AVT and alcohol abstinence).The criteria for the above stratification is detailed in bold in the Additional file 1. Covariates included in the multivariate logistic regressions were identified through univariate analysis. Statistical data were expressed as the median and 25^th^, 75th percentiles or as percentage as appropriate. Kruskal–Wallis H test along with Bonferroni correction was used to compare quantitative variables between groups. Chi square (*X*^2^) test was used to compare categorical variables. All statistical tests were two-sided; *P* values below 0.05 were considered statistically significant. All data were analyzed using SPSS 25.0 (Armonk, NY: IBM corporation).

## Results

A total of 25,782 cases of liver cirrhosis diagnosed in our hospital from January 2010 to December 2019 were collected. Cases of congenital biliary atresia (35 cases), Wilson disease (28 cases), autoimmune liver disease (842 cases), cases with two or more etiologies other than ALD + HBV or ALD + HCV (930 cases), cases with incomplete data, unconfirmed or unknown cirrhosis etiology (1660) were all excluded from this study. The remaining cases (22,287 cases) were divided into five groups according to their etiologies: ALD group, HBV group, ALD + HBV group, HCV group and ALD + HCV group. The changing trend of the proportion of each etiology over the ten-year period is depicted in Additional file 1: Figure S1. HBV was found to be the major cause of cirrhosis, with an annual proportion fluctuating around 80% over the ten-year period. The median age of the studied population was 52 [44, 60] years, with a total of 18,744 males (84.1%, median age 51 [43, 59] years) and 3543 females (15.9%, median age 58 [50, 65] years). The difference in age between male and female was statistically significant (*P* < 0.001). The age-sex distribution of the studied population is displayed in Additional file 1: Figure S2. Among the five groups, the HBV group represented the highest proportion of all cirrhosis cases (18,079 cases, 81.1%, median age 52 [44, 61] years), followed by the ALD group (1652 cases, 7.4%, median age 52 [46, 59] years), ALD + HBV group (1594 cases, 7.2%, median age 53 [46, 60] years), HCV group (682 cases, 3.1%, median age 55 [47, 64] years) and ALD + HCV group (280 cases, 1.3%, median age 46 [41, 53] years). The differences in age and sex between the five groups were statistically significant, *P* < 0.001, see Table 1. Other characteristics such as patient’s main chief complaints on admission, duration of alcoholism, alcohol abstinent state, use of antiviral therapy, etc., is summarized in Additional file 1: Table S1.

**Table 1:** Liver function characteristics of the studied groups

Parameters	ALD n = 1652	HBV n = 18,079	HCV n = 682	ALD + HBV n = 1594	ALD + HCV n = 280	*P* value
Sex: male	1611 (97.5%)	14,914 (82.5%)^a^	391 (57.3%)^a,b,d^	1570 (98.5%)^b^	258 (92.1%)^a,b,c,d^	< 0.001
Age (years): median (P_25,_ P_75_)	52 (46, 59)	52 (44, 61)	55 (47, 64)^a,b,d^	53 (46, 60)	46 (41, 53)^a,c,d^	< 0.001
Decompensation	975 (59.0)	7039 (38.9)^a^	221 (32.4)^a,b^	888 (55.7)^b,c^	152 (54.3)^b,c^	< 0.001
MCV (fl)	93.7 (84.4, 101.1)	90.1 (84.2, 94.9)^a^	91.3 (85.4, 95.8)^a,d^	92.6 (85.9, 98.3)^b^	93.8 (85.6, 100)^b,c,d^	< 0.001
ALT (U/L)	34 (22, 55)	44 (28, 85)^a^	47 (28, 79)^a,d^	51 (31, 94)^a,b^	43 (29, 76)^a,b,d^	< 0.001
AST (U/L)	65 (40, 108)	58 (35, 117)	63 (38, 99)^d^	83 (47, 158)^a,b^	79 (50, 141)^a,b,c^	< 0.001
AST/ALT	1.9 (1.3, 2.7)	1.3 (0.9, 1.8)^a^	1.3 (1.0, 1.8)^a,d^	1.5 (1.1, 2.2)^a,b^	1.7 (1.3, 2.5)^b,c,d^	< 0.001
GGT (U/L)	169 (66, 364)	72 (37, 153)^a^	55 (32, 114)^a,b,d^	137 (65, 274)^b^	119 (58, 229)^a,b,c^	< 0.001
MELD score	11.4 (7.0, 17.7)	9.2 (5.7, 15.2)^a^	7.3 (4.3, 11.2)^a,b,d^	11.1 (6.7, 17.7)^b^	10.0 (7.0, 14.7)^c^	< 0.001
MELD ≥ 18	389 (24.3%)	3257 (18.5%)^a^	50 (7.6%)^a,b,d^	379 (24.1%)^b^	36 (13.1%)^a,d^	< 0.001
GAHS	7.0 (6.0, 8.0)	7.0 (6.0, 7.0)^a^	6.0 (6.0, 7.0)^a,b,d^	7.0 (6.0, 8.0)^b^	6.0 (5.0, 7.0)^a,b,d^	< 0.001
GAHS ≥ 9	238 (14.8%)	2014 (11.5%)^a^	22 (3.3%)^a,b,d^	222 (14.1%)^b^	20 (7.3%)^a,d^	< 0.001
MDF score	23.7 (13.3, 41.0)	18.8 (11.0, 34.6)^a^	16.1 (10.7, 25.2)^a,b,d^	22.4 (12.6, 40.3)^a,b^	20.3 (13.3, 33.5)^c^	< 0.001
MDF ≥ 32	579 (36.1%)	4883 (27.7%)^a^	102 (15.5%)^a,b,d^	430 (33.7%)^b^	73 (26.5%)^a,c^	< 0.001
Child A	413 (25.7%)	8044 (45.7%)^a^	351 (53.4%)^a,b,d^	489 (31.1%)^a,b^	83 (30.3%)^b,c,d^	< 0.001
Child B	758 (47.3%)	6271 (40.9%)^a^	242 (36.8%)^a,b,d^	644 (40.9%)^a,b^	141 (51.5%)^b,c,d^	< 0.001
Child C	433 (27.0%)	3304 (18.8%)^a^	64 (9.7%)^a,b,d^	440 (28.0%)^a,b^	50 (18.2%)^b,c,d^	< 0.001

Values are expressed in median (25th, 75th percentiles) unless indicated otherwise

MCV, Mean Corpuscular Volume; ALT, alanine aminotransferase; AST, aspartate aminotransferase; GGT, Gamma-glutamyl transferase; MELD score, Model for End-stage Liver Disease; GAHS, Glasgow Alcoholic Hepatitis Score; MDS, Maddrey’s Discriminant function Scale. MELD ≥ 18, GAHS ≥ 9 and MDF ≥ 32 represent proportion of patients with MELD, GAHS and MDF greater or equal to 18, 9 and 32 respectively. Child A, B, C represents respectively proportion of Child–Pugh A, B and C classification patients with complete data

^a^Means value statistically significant compared to that in ALD; ^b^means value statistically significant compared to that in HBV; ^c^means value statistically significant compared to that in HCV; ^d^means value statistically significant compared to that in HBV + ALD. Bonferroni correction was used for “*P*” during pairwise comparisons

### Characteristics of the laboratory data

Differences in the laboratory data between the five groups are detailed in Table 1. Serum level of MCV was more elevated in the ALD group compared to that in both HBV group and HCV group. Moreover, MCV appeared higher in both ALD + HBV and ALD + HCV groups compared to HBV group and to HCV group respectively (*P* < 0.001). AST/ALT was higher in the ALD group compared to the HBV group and to the HCV group. This ratio was more elevated in ALD + HBV group compared to that in HBV (1.5 [1.1, 2.2] vs 1.3 [0.9, 1.8], *P* < 0.05) and also in ALD + HCV compared to HCV (1.7 [1.3, 2.5] vs 1.3 [1.0, 1.8], *P* < 0.05). A similar trend was observed while comparing GGT levels between ALD, HBV and HCV groups, between ALD + HBV and HBV and also between ALD + HCV and HCV groups. ALD patients presented a higher MELD score compared to HBV patients and to HCV patients (11.4 [7.0, 17.7] vs 9.2 [5.7, 15.2] vs 7.3 [4.3, 11.2] respectively). Similarly, the percentage of patients with MELD score ≥ 18 was higher in the ALD group compared to that in the HBV and HCV groups (24.3% vs 18.5% vs 7.3% respectively, *P* < 0.05). Furthermore, the median MELD score of the ALD + HBV group surpassed that of HBV group (11.1 [6.7, 17.7] vs 9.2 [5.7, 15.2], *P* < 0.05), so did the MELD score in ALD + HCV group compared to that in HCV group (10.0 [7.0, 14.7] vs 7.3 [4.3, 11.2], *P* < 0.05). Likewise, the proportions of patients with MELD score ≥ 18 were higher in the ALD + HBV group compared to that in the HBV group (24.1% vs 18.5%, *P* < 0.05) and also in the ALD + HCV group compared to the HCV group (13.1% vs 7.6%, *P* < 0.05). Similar trends were observed assessing GAHS, MDF and Child–Pugh classification between the five groups. In addition, ALD patients presented with higher proportion of decompensation complaints compared to HBV and to HCV patients (59.0% vs 38.9% vs 32.4%, *P* < 0.001). Patients with ALD + HBV had higher proportion of decompensation symptoms compared to HBV patients (55.7% vs 38.9%, *P* < 0.001); so were the ALD + HCV patients compared to the HCV patients (54.3% vs 32.4%, *P* < 0.001).

### Assessment of different liver cirrhosis complications between the groups

HCC was less frequent in the ALD group (10.3%) compared to the HBV group (42.2%) and to the HCV group (20.8%), *P* < 0.001. Moreover, HCC was more prevalent in the ALD + HBV group compared to the HBV group (52.2% vs 42.2%, *P* < 0.001), and also in the ALD + HCV group compared to the HCV group (34.3% vs 20.8%, *P* < 0.001), see Table 2. A multivariate logistic regression estimated the risk of HCC in ALD + HBV group at 2.01 time that of HBV group (95% CI 1.58–2.55, *P* < 0.001) after adjusting for confounders. Similarly, the risk of HCC in ALD + HCV group was estimated to be 2.61 times that of HCV group (95% CI 1.63–4.18, *P* < 0.001). Other independent predictors of HCC are displayed in Table 3 and Additional file 1: Table S2.

**Table 2:** Proportion of cirrhosis complications in the studied groups

Complications	ALD n (%)	HBV n (%)	HCV n (%)	ALD + HBV n (%)	ALD + HCV n (%)	*P* value
HCC	170 (10.3)	7622 (42.2)^a^	142 (20.8)^a,b,d^	832 (52.2)^a,b^	96 (34.3)^a,c,d^	< 0.001
Infection	553 (33.5)	4184 (23.1)^a^	129 (18.9)^a,d^	486 (30.5)^b^	79 (28.2)^c^	< 0.001
Ascites	892 (54.0)	7835 (43.3)^a^	252 (37.0)^a,b,d^	879 (55.1)^b^	144 (51.4)^c^	< 0.001
HE	127 (7.7)	947 (5.2)^a^	20 (2.9)^a,d^	104 (6.5)	14 (5.0)	< 0.001
Thrombus	102 (6.2)	1040 (5.8)	35 (5.1)	80 (5.0)	12 (4.3)	0.461
EGV^†^	813 (49.6)	6612 (38.3)^a^	266 (41.1)^b^	707 (45.5)^a,b^	129 (46.1)^b^	0.001
EGVB^‡^	318 (39.1)	1990 (30.1)^a^	87 (32.7)	306 (43.3)^b^	66 (51.2)^c^	< 0.001
HRS	49 (3.0)	276 (1.5)^a^	8 (1.2)	35 (2.2)	3 (1.1)	< 0.001

HCC, hepatocellular carcinoma; HE, hepatic encephalopathy; EGV, esophageal gastric varices; EGVB, esophageal gastric variceal bleeding, HRS, hepatorenal syndrome

^a^Means value statistically significant compared to that in ALD; ^b^means value statistically significant compared to that in HBV; ^c^means value statistically significant compared to that in HCV; ^d^means value statistically significant compared to that in HBV + ALD. Bonferroni correction was used for “*P*” during pairwise comparisons

^†^The proportion of EGV was calculated by dividing the number of patients reported with EGV by the number of patients who underwent upper GI endoscopy or other imaging examination such as CT or MR, capable of detecting EGV; ^‡^The proportion of EGVB was calculated by dividing number of patients reported with EGVB by the number of confirmed EGV patients

**Table 3:** Univariate and multivariate logistic regression evaluating factors associated with HCC in ALD, HBV and ALD + HBV patients

Factors	Without HCC	With HCC	ULR	MLR	
	n (%)	n (%)	*P* value	OR (95% CI)	*P* value
Sex (male)	10,298 (81.1)	7797 (90.4)	< 0.001	2.55 (1.95–3.34)	< 0.001
Age (> 50y)	6861 (54.0)	5481 (63.6)	< 0.001	1.79 (1.54–2.09)	< 0.001
Etiologies			< 0.001		
HBV	10,457 (82.3)	7622 (88.4)		1	
ALD	1482 (11.7)	170 (2.0)		0.18 (0.12–0.26)	< 0.001
ALD + HBV	762 (6.0)	832 (9.6)		2.01 (1.58–2.55)	< 0.001
T2DM	1726 (14.4)	757 (9.2)	< 0.001	0.41 (0.32–0.52)	< 0.001
Decomp. Sx	6657 (52.4)	2245 (26.0)	< 0.001	0.59 (0.48–0.73)	< 0.001
Infection	3906 (30.8)	1317 (15.3)	< 0.001	0.70 (0.58–0.84)	< 0.001
Ascites	6153 (48.4)	3453 (40.0)	< 0.001	1.85 (1.54–2.22)	< 0.001
HE	1014 (8.0)	164 (1.9)	< 0.001	0.62 (0.43–0.89)	0.010
Thrombus	670 (5.3)	552 (6.4)	0.001	1.47 (1.09–1.98)	0.011
HRS	282 (2.2)	78 (0.90)	< 0.001		
EGVB	1801 (34.1)	813 (28.5)	< 0.001	1.46 (1.19–1.80)	< 0.001
Child classification			< 0001		
A	4059 (32.9)	4887 (57.8)		1	
B	4914 (39.8)	2759 (32.6)		0.66 (0.54–0.81)	< 0.001
C	3363 (27.3)	814 (9.6)		0.41 (0.31–0.54)	< 0.001
MCV > 100	1758 (13.9)	673 (7.9)	< 0.001		
ALT > 40	6770 (53.8)	4617 (54.0)	0.856		
AST > 40	6584 (92.8)	4287 (90.1)	< 0.001		
GGT > 60	4753 (67.7)	4012 (85.0)	< 0.001	4.00 (3.31–4.82)	< 0.001

ULR, univariate logistic regression; MLR, multivariate logistic regression; HBV, hepatitis B virus; ALD, alcohol-induced liver disease; ALD + HBV, co-existing ALD and HBV; HCC, Hepatocellular carcinoma; T2DM, type 2 diabetes mellitus; Decomp. Sx., decompensation symptoms and signs; HE, Hepatic Encephalopathy; EGVB, Esophageal gastric variceal bleeding, HRS, Hepatorenal Syndrome; T2DM, Type 2 diabetes mellitus; MCV > 100, ALT > 40, AST > 40, GGT > 60 represent proportion of patients with mean corpuscular volume, alanine aminotransferase, aspartate aminotransferase, Gamma-glutamyl transferase greater than 100, 40, 40, and 60 respectively

The ALD group registered a higher proportion of EGVB compared to HBV group (39.1% vs 30.1%, *P* < 0.001). This difference was non-significant comparing ALD group to HCV group (39.1% vs 32.7%, *P* > 0.05). The proportion of EGVB was also significantly higher in ALD + HBV group than that in HBV group (43.3% vs 30.1%, *P* < 0.001); see Table 2. A multivariate logistic regression analysis evaluated the risk of EGVB in ALD + HBV group to be 1.74 times that of HBV group (95% CI 1.30–2.33, *P* < 0.001) after adjusting for confounders. Similarly, EGVB was more frequent in ALD + HCV group than in the HCV group (51.2% vs 32.7%). Moreover, the risk of EGVB in the ALD + HCV patients was 2.91 times that in the HCV patients (95% CI 1.08–7.86, *P* = 0.035). Other independent predictors for EGVB are displayed in Table 4 and Additional file 1: Table S3. Other liver cirrhosis complications such as infection, ascites, HE, and HRS were all significantly more frequent in the ALD group (33.5%, 54.0%, 7.7%, 3.0% respectively) compared to the HBV group (23.1%, 43.3%, 5.2%, 1.5% respectively) and to the HCV group (18.9%, 37.0%, 2.9%, 1.2% respectively). Furthermore, apart from HRS, these complications were significantly more common in the ALD + HBV group compared to the HBV group, and also in the ALD + HCV group compared to the HCV group, see Table 2.

**Table 4:** Univariate and multivariate logistic regression evaluating factors associated with EGVB in ALD, HBV and ALD + HBV patients

Factors	Without EGVB	With EGVB	ULR	MLR	
	n (%)	n (%)	*P* value	OR (95% CI)	*P* value
Sex (male)	4717 (85.5)	2282 (87.3)	0.026		
Age (> 50y)	3288 (59.6)	1425 (54.5)	< 0.001		
Etiologies			< 0.001		
HBV	4622 (83.8)	1990 (76.1)		1	
ALD	495 (9.0)	318 (12.2)		1.14 (0.81–1.59)	0.451
ALD + HBV	401 (7.3)	306 (11.7)		1.74 (1.30–2.33)	< 0.001
T2DM	644 (11.7)	252 (10.2)	0.060		
Decomp. Sx	1914 (34.7)	2614 (100)	< 0.001		
Infection	1060 (19.2)	690 (26.4)	< 0.001		
Ascites	2835 (51.4)	1403 (53.7)	0.053	0.51 (0.41–0.64)	< 0.001
HE	226 (4.1)	218 (8.3)	< 0.001	1.44 (1.07–1.95)	0.018
Thrombus	449 (8.1)	276 (10.6)	< 0.001	1.35 (1.01–1.79)	0.039
HCC	2043 (37.0)	813 (31.1)	< 0.001	1.46 (1.19–1.78)	< 0.001
HRS	45 (0.80)	72 (2.8)	< 0.001	2.58 (1.54–4.32)	< 0.001
Child			< 0.001		
A	2131 (39.2)	911 (36.4)		1	
B	2317 (42.6)	1043 (41.7)		0.12 (0.08–0.19)	< 0.001
C	988 (18.2)	549 (21.9)		0.10 (0.07–0.16)	
MCV > 100	753 (13.7)	255 (9.8)	< 0.001	0.69 (0.54–0.90)	0.006
ALT > 40	2650 (48.3)	1120 (43.3)	< 0.001		
AST > 40	2500 (94.3)	1117 (92.4)	0.023		
GGT > 60	1952 (74.1)	816 (69.4)	0.003		

ULR, univariate logistic regression; MLR, multivariate logistic regression; HBV, hepatitis B virus; ALD, alcohol-induced liver disease; ALD + HBV, co-existing ALD and HBV; HCC, Hepatocellular carcinoma; T2DM, type 2 diabetes mellitus; Decomp. Sx., decompensation symptoms and signs; HE, Hepatic Encephalopathy; EGVB, Esophageal gastric variceal bleeding, HRS: Hepatorenal Syndrome; MCV > 100, ALT > 40, AST > 40, GGT > 60 represent proportion of patients with mean corpuscular volume, alanine aminotransferase, aspartate aminotransferase, Gamma-glutamyl transferase greater than 100, 40, 40, and 60 respectively

### Differences in the proportion of HCC and EGVB between sexes

**Fig. 1:**
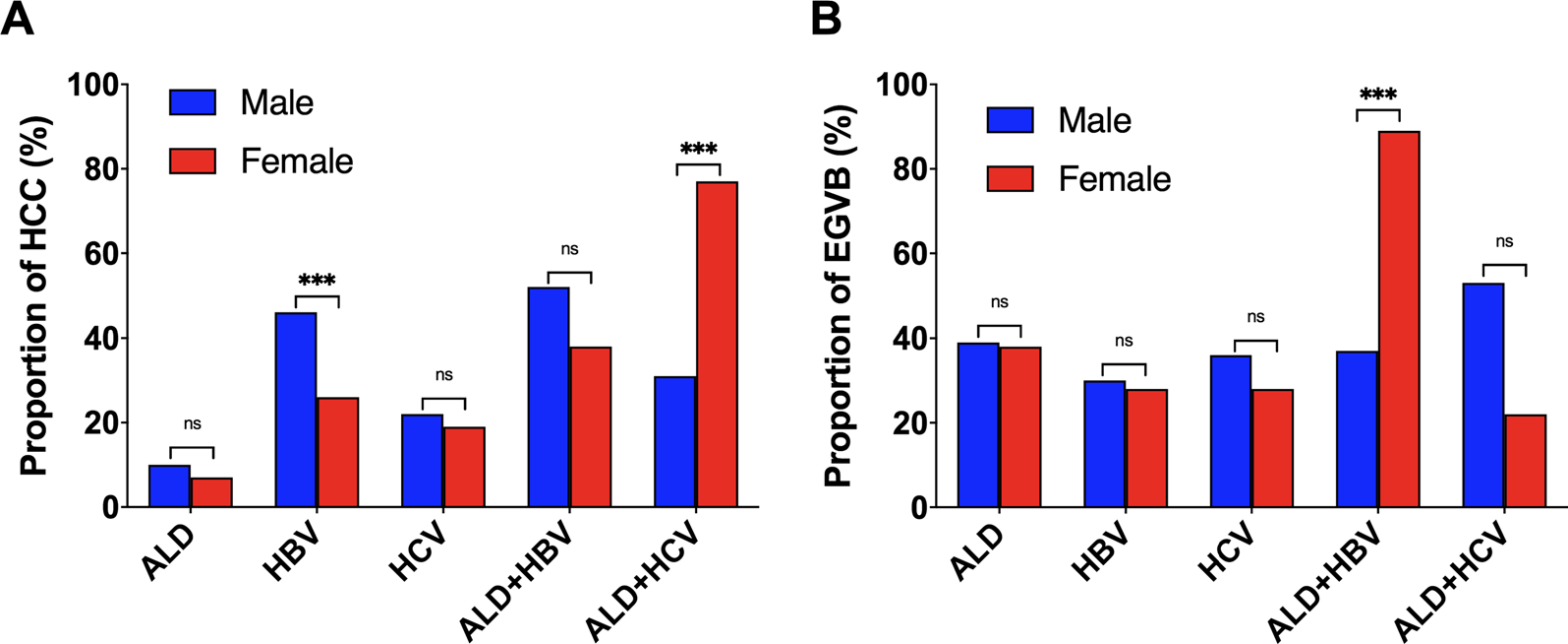
Proportion of HCC (**A**) and EGVB (**B**) according to sex in different groups. ALD, alcohol-induced liver disease; HBV, Hepatitis B virus; HCV, hepatitis C virus; ALD + HBV, co-existing ALD and HBV; ALD + HCV, co-existing ALD and HCV; HCC, Hepatocellular carcinoma; EGVB, Esophageal gastric variceal bleeding. “***” indicates *P* < 0.001, “ns” means non-significant (*P* > 0.05)

In the ALD group, the proportion of HCC and EGVB were similar between males and females (HCC: 10.4% vs 7.3% *P* = 0.526; EGVB: 39.1% vs 38.9%, *P* = 0.984). In the HBV group, HCC was more prevalent in males (45.6% vs 25.8%, *P* < 0.001), whereas the prevalence of EGVB appeared similar between both sexes (*P* = 0.122). Contrary to HBV group, in the ALD + HBV group, HCC was similarly frequent between males and females (52.4% vs 37.5%, *P* = 0.146). However, the proportion of EGVB in females surpassed drastically that in males (36.8% vs 88.9%, *P* < 0.001). In the HCV group, similar rate of HCC and EGVB was found between males and females (HCC: *P* = 0.287; EGVB: *P* = 0.153). On the other hand, in the ALD + HCV group the proportion of HCC was remarkably higher in females (30.6% vs 77.3%, *P* < 0.001). However, EGVB was similarly frequent in both sexes (*P* = 0.072). The results of these comparisons are depicted in Fig. 1.

### Effect of alcohol abstinence on the proportions of HCC and EGVB in ALD, ALD + HBV and ALD + HCV

ALD, ALD + HBV and ALD + HCV groups were each divided into four subgroups according to the duration of alcohol abstinence: “no abstinence”, “abstinent less than five years”, “abstinent five to ten years” and “abstinent more than ten years”. A linear-by-linear association test from chi square analysis between duration of abstinence and different groups (ALD, ALD + HBV, ALD + HCV) revealed that the proportion of HCC decreased with the duration of alcohol abstinence (linear-by-linear association overall *P* < 0.001 in the three groups), see Additional file 1: Figure S3A. Similarly, a multivariate logistic regression analysis showed a downward trend in the risk of HCC with the duration of abstinence in the three groups. In patients with an abstinence period greater than ten years, this risk was estimated at 0.27 (95% CI 0.14–0.50, *P* < 0.001) in ALD group, 0.01 (95% CI 0.00–0.03, *P* < 0.001) in ALD + HBV group and at 0.10 (95% CI 0.01–0.82, *P* = 0.032) in ALD + HCV group, after adjusting confounders, and using non abstinent patients as reference, as summarized in Table 5, and detailed in Additional file 1: tables S5, S7 and S9.

**Table 5:** Relation between alcohol abstinence and risk of HCC in ALD, ALD + HBV and ALD + HCV patients

	Without HCC	With HCC	ULR	MLR	
	n (%)	n (%)	*P* value	OR(95% CI)	*P* value
ALD					
			0.001		
No abstinence	942 (63.6)	131 (77.1)		1	
Abstinent < 5 years	156 (10.5)	18 (10.6)		0.71 (0.40–1.24)	0.226
Abstinent 5–10 years	136 (9.20)	9 (5.30)		0.37 (0.18–0.77)	0.008
Abstinent > 10 years	248 (16.7)	12 (7.10)		0.27 (0.14–0.50)	< 0.001
ALD + HBV					
			< 0.001		
No abstinence	370 (48.6)	684 (82.2)		1	
Abstinent < 5 years	163 (21.4)	98 (11.8)		0.10 (0.04–0.24)	< 0.001
Abstinent 5–10 years	111 (14.6)	34 (4.1)		0.01 (0.00–0.04)	< 0.001
Abstinent > 10 years	118 (15.5)	16 (1.9)		0.01 (0.00–0.03)	< 0.001
ALD + HCV					
			< 0.001		
No abstinence	119 (64.7)	85 (89.5)		1	
Abstinent < 5 years	38 (20.5)	7 (7.4)		0.26 (0.10–0.68)	0.010
Abstinent 5–10 years	15 (8.1)	2 (2.1)		0.17 (0.03–0.84)	0.037
Abstinent > 10 years	13 (7.1)	1 (1.1)		0.10 (0.01–0.82)	0.047

ULR, univariate logistic regression; MLR, multivariate logistic regression; HCC, Hepatocellular carcinoma; EGVB, Esophageal gastric variceal bleeding. The logistic regression analysis in ALD group was adjusted for age, alcoholism duration, presence of decompensation, MCV, ALT, presence of infection, hepatic encephalopathy, thrombus, and child classification. In ALD + HBV group, the analysis was adjusted for age, alcoholism duration, MCV, GGT, presence of diabetes, presence of decompensation, infection, HE, thrombus, HRS, EGVB and child classification. In ALD + HCV group, the analysis was adjusted for age, sex, alcoholism duration, presence of decompensation, thrombus, infections and MCV. See Additional file: Tables S5, S7 and S9 for detailed results

On the other hand, similar downward trend in the proportion of EGVB was observed with the duration of alcohol abstinence in ALD, ALD + HBV and ALD + HCV group (linear-by-linear association overall *P* < 0.001 in the three groups), see Additional file 1: Figure S3B. This was confirmed in the multivariate logistic regression analysis, which revealed a lower risk of EGVB in patients abstinent from alcohol for more than ten years (OR = 0.06, 95% CI 0.01–0.45, *P* = 0.007 in ALD; OR = 0.08, 95% CI 0.04–0.16, *P* < 0.001 in ALD + HBV; OR = 0.03, 95% CI 0.00–0.51, *P* = 0.015 in ALD + HCV), as summarized in Additional file 1: Table S4 and detailed in Additional file 1: Tables S6, S8 and S10.

### Influence of antiviral treatment on the proportions of HCC and EGVB in HBV, ALD + HBV, HCV and ALD + HCV

After stratifying each group’s patients into subgroups based on their antiviral treatment status (in HBV and in HCV groups) and based on AVT status coupled with the duration of alcohol abstinence (in the ALD + HBV and the ALD + HCV groups), we found that in the HBV group, patients with AVT had significantly lower risk of presenting with HCC and EGVB compared to those without AVT (HCC: OR = 0.31, 95% CI 0.26–0.36, *P* < 0.001; EGVB: OR = 0.39, 95% CI 0.29–0.52, *P* < 0.001). Similarly, in the HCV group, the risk of HCC and that of EGVB was lower in the AVT subgroup compared to that in the no AVT subgroup (HCC: OR = 0.50, 95% CI 0.34–0.73, *P* < 0.001; EGVB: OR = 0.34, 95% CI 0.20–0.57, *P* < 0.001). In the ALD + HBV group, patients with both treatments (abstinence and AVT) had lower risk of HCC (OR = 0.10, 95% CI 0.05–0.20, *P* < 0.001) and of EGVB (OR = 0.17, 95% CI 0.06–0.45, *P* < 0.001) compared to those without any treatment (no abstinence and no AVT). Moreover, patients with only one kind of treatment (either AVT + no abstinence, or no AVT + abstinence) had lower risk of HCC and EGVB compared to those without any treatment. However, these values were still higher than that in the AVT + abstinence subgroup. Similar trend was observed while assessing the risk of HCC and EGVB in the different subgroups of the ALD + HCV patients, see Tables 6 and 7.

**Table 6:** Relation between antiviral treatment and the odds of HCC in HBV, ALD + HBV and ALD + HCV patients

Variables	n (%)	OR	95% CI	*P* value
HBV				
No AVT	4246/9620 (44.1)	1		
AVT	168/863 (19.5)	0.31	0.26–0.36	< 0.001
HCV				
No AVT	71/251 (28.3)	1		
AVT	71/431 (16.5)	0.50	0.34–0.73	< 0.001
ALD + HBV				
No AVT + no abst	431/592 (72.8)	1		
No AVT + abst	71/275 (25.8)	0.13	0.10–0.18	< 0.001
AVT + no abst	32/79 (40.5)	0.25	0.16–0.41	< 0.001
AVT + abst	13/60 (21.7)	0.10	0.05–0.20	< 0.001
ALD + HCV				
No AVT + no abst	48/105 (45.7)	1		
No AVT + abst	8/28 (28.6)	0.47	0.19–1.17	0.107
AVT + no abst	33/87 (37.9)	0.73	0.41–1.29	0.278
AVT + abst	6/60 (10.0)	0.13	0.05–0.33	< 0.001

HCC, Hepatocellular carcinoma; AVT, antiviral treatment; abst, alcohol abstinence; HBV, hepatitis B virus; HCV, hepatitis C virus; ALD, alcohol-induced liver disease; ALD + HCV, co-existing ALD and HCV. Only patients with information on AVT were included in this analysis

**Table 7:** Relation between antiviral treatment and the odds of EGVB in HBV, ALD + HBV and ALD + HCV patients

Variables	n (%)	OR	95% CI	*P* value
HBV				
No AVT	985/3302 (29.8)	1		
AVT	57/399 (14.3)	0.39	0.29–0.52	**< **0.001
HCV				
No AVT	51/109 (46.8)	1		
AVT	36/157 (22.9)	0.34	0.20–0.57	< 0.001
ALD + HBV				
No AVT + no abst	100/194 (51.5)	1		
No AVT + abst	41/158 (25.9)	0.33	0.21–0.52	< 0.001
AVT + no abst	9/32 (28.1)	0.37	0.16–0.84	0.017
AVT + abst	5/33 (15.2)	0.17	0.06–0.45	< 0.001
ALD + HCV				
No AVT + no abst	25/36 (69.4)	1		
No AVT + abst	5/18 (27.8)	0.17	0.05–0.59	0.005
AVT + no abst	32/51 (62.7)	0.74	0.30–1.84	0.518
AVT + abst	4/25 (16.0)	0.08	0.02–0.30	< 0.001

Only patients with information on AVT were included in this analysis

EGVB, esophageal gastric variceal bleeding; AVT, antiviral treatment; abst, alcohol abstinence; HBV, hepatitis B virus; HCV, hepatitis C virus; ALD, alcohol-induced liver disease; ALD + HCV, co-existing ALD and HCV.

## Discussion

Owing to the reputation of our center in the diagnosis and management of liver diseases, the findings of this research can effectively reflect the situation of coexisting ALD and viral hepatitis in southern China. Our study successfully showed the exacerbation of liver impairment and the increase in the proportions of liver cirrhosis complications among coexisting ALD and viral hepatitis patients.

In this study, over 80% of the enrolled patients were diagnosed with HBV-induced liver cirrhosis, placing HBV as the leading cause of liver cirrhosis in our hospital. This finding is consistent with the situation in other Asia–Pacific region countries [10, 11]. Carbohydrate deficient transferrin, ethyl glucuronide, phosphatidyl ethanol, etc. had shown their importance in the diagnosis of ALD and in the evaluation of alcohol abstinence[4], however, elevated blood levels of MCV, GGT and AST/ALT remained the most commonly used laboratory characteristics in ALD [12, 13]. These characteristics were also reflected in our study, with levels of MCV, GGT and AST/ALT significantly higher in the ALD group compared to HCV and to HBV groups. Furthermore, these values were worse in viral hepatic cirrhosis patients with heavy alcohol consumption (ALD + HBV and ALD + HCV). To our knowledge there has been no previous report on the synergic effect of alcohol and hepatitis virus on these laboratory markers. Our findings can therefore help clinicians suspect the presence of heavy alcohol use in viral hepatic cirrhosis patients with unclear history of alcohol intake.

Disease severity was compared between ALD, HBV and HCV, between ALD + HBV and HBV, and also between ALD + HCV and HCV. On a laboratory scale, the ALD patients presented with more severe liver impairment compared to HBV and to HCV patients, with the highest proportion of patients with MELD ≥ 18, GAHS ≥ 9, MDS ≥ 32 and child C. These findings are in line with that of Astrid Marot et al. [14] who concluded in their study that ALD patients had worse liver function at admission compared to other liver cirrhosis patients. In our study, the median value of the above-mentioned assessment scores, and the proportion of poor prognosis patients (MELD ≥ 18, GAHS ≥ 9, MDS ≥ 32, Child C) were all higher in coexisting ALD and viral hepatitis compared to the corresponding viral hepatitis group (ALD + HBV vs HBV and ALD + HCV vs HCV). This edifies the negative effect of alcohol on the liver function of HBV and HCV patients. Possible explanations that had been given in previous literatures are: the enhancement of hepatitis viral replication by alcohol [15, 16], delayed hepatitis B “e” antigen loss [17], increased hepatocyte toxicity, increased oxidative stress and more weakened host immune response [18, 19].

In view of the difference in the pathogenesis of different etiologies of liver cirrhosis, it is expected that the decompensation pattern of liver cirrhosis may differ according to the etiologies. In our study, HCC was less prevalent in the ALD group compared to HBV and to HCV groups. This finding is identical to that of Okada et al. [20] and that of Astrid Marot et al. [14]. Apart from HCC, other liver cirrhosis complications were all more prevalent in the ALD group compared to HBV and HCV group. This high frequency of cirrhosis complications in ALD patients was also pointed out in a former study on Danish population [21]. Moreover, some researches had proven the role of alcohol in the acceleration of liver cirrhosis decompensation and the worsening of survival in HCV patients [22, 23]. This is in accordance with our study which revealed that the proportion of HCC was significantly higher in ALD + HCV patients compared to that in HCV. Khan et al. [24] also noticed a rise of 1.5 to 2.5 fold in the risk of HCC while assessing the progression of HCV-related liver disease and the risk of HCC onset in moderate and heavy Japanese drinkers. Their study also showed that cirrhosis complications such as EGVB, ascites and hepatic encephalopathy were more frequent in heavy alcohol drinkers with HCV than in alcohol abstinent HCV Japanese patients. This supports findings of our study where we noticed that complications such as infection, ascites, HE, thrombus formation, HRS, and EGVB were all more prevalent in ALD + HCV group compared to HCV group. Suggested explanation was that during the natural evolution of HCV-related liver disease, repeated regeneration of the hepatocytes due to persistent liver injury by HCV may cause hepatocyte DNA to become susceptible to mutagenesis, resulting in gene instability [25]. Furthermore, ethanol induces enzymatic activation for the conversion of procarcinogens into carcinogens and consequential induction of hepatic neoplasm [26, 27]. Likewise, recent researches had mentioned the negative impact of alcohol on the decompensation of HBV-induced liver cirrhosis patients. Our study, which was held in a center with one of the highest cases of HBV management in southern China, had revealed that HCC was far more frequent in HBV patients compared to ALD patients. Moreover, the ALD + HBV group had noticed higher proportion of HCC compared to the HBV group. These findings match those of Larkin et al. [15] in their study on the impact of ethanol on HBV gene expression and replication in transgenic mice. Similar observations were made clinically in several other studies during the last decades [23, 28–30].

In general, women are known to have lower rate of liver cirrhosis decompensation and malignant tumors compared to men [31]. However, studies showed that women with ALD have more rapid progression to fibrosis and HCC compared to men [32, 33]. This is partly due to higher endotoxin levels, increased gut permeability to endotoxins noted in females, and also to the important role of estrogen in the activation of liver Kupffer cells [34, 35]. Controversially, in HCV-induced liver cirrhosis, estrogen has been shown to inhibit stellate cells, which are responsible for fibrogenesis in the liver [36], thus less decompensation in liver function. Studies on HBV-induced HCC are scanty, however it has been reported that the risk of HCC is higher in males with HBV compared to females [37]. This is also the case in our study where the proportion of HBV-induced HCC in males was almost double that in females. Researches on the difference in the prevalence of EGVB between sexes are scarce. To our knowledge, there was no previous literature comparing the influence of alcohol on the prevalence of HCC and EGVB between sexes in HBV or HCV patients. In our study, we found that the proportion of HCC was similar between both sexes in HCV group, however this proportion became significantly higher in females in ALD + HCV group. On the other hand, EGVB which was in similar proportion between males and females in HBV became more prominent in females in ALD + HBV group. Therefore, more surveillance should be given to females with ALD + HBV in the follow-up of esophageal gastric varices. Similarly, females with ALD + HCV should be more often screened for HCC during their follow-up. However, a prospective study on a larger population, with both pre-menopause and post menopause women is needed to validate these findings.

Studies done on the effect of alcohol abstinence on ALD all over the world are not unanimous. In our study, patients with ALD, ALD + HBV and ALD + HCV all noticed a significant reduction of the proportion of HCC and EGVB following alcohol abstinence. This reduction appeared to be more remarkable with longer duration of abstinence. These findings are in accordance with some previously published researches on the positive long-term effect of alcohol abstinence [38–41]. Moreover, Verrill et al. [42] in their study found that patients with more severe liver cirrhosis had greater benefit from alcohol abstinence compared to those with relatively mild cases of cirrhosis. Controversially, Evangelos et al.[43] in their study noticed that cirrhotic patients who had abstained from alcohol showed more severe liver cirrhosis expressed as higher MELD score compared to those who had not abstained. Moreover, they found no difference in the prevalence of HCC between the abstinent and the non-abstinent patients. Similarly, few more studies had rejected the negative effect of alcohol on the prognosis of ALD patients [44, 45]. Nevertheless, Alcohol abstinence is undeniably the cornerstone in the management of ALD. On the other hand, whether the undeniable benefits of AVT had led to a reduction of HCC burden and prevalence of EGVB is still on debate. In our study, the use of AVT has shown a decrease in the proportion of HCC and EGVB in both HBV and HCV patients. In the ALD + HBV and the ALD + HCV patients, those with both AVT and alcohol abstinence appear to have lower risk of HCC and EGVB compared to those without any treatment and also compared to those with only one kind of treatment (AVT + no abstinence and no AVT + abstinent). These findings which goes along with those of Chiang et al. [46] need to be confirmed with a multicentered prospective study with limited influencing cofactors.

In conclusion, Alcohol increased significantly the severity of liver function impairment and the prevalence of liver cirrhosis complications particularly HCC and EGVB in hepatitis virus-induced liver cirrhosis patients (HBV and HCV). Remarkably, long-term abstinence from alcohol coupled with efficient antiviral treatment effectively decreased the prevalence of HCC and EGVB in these populations, thus the importance of stressing not only on the importance of antiviral treatment in patients with coexisting ALD and viral cirrhosis, but also on long term alcohol abstinence.

However, our study has some limitations that are worth mentioning. Indeed, due to the retrospective nature of this study, biases such as recall bias from the patients, misclassification bias, etc., could be expected. However, a thorough reexamination of patients’ admission file was carried out to reduce the occurrence of these biases. Even though our center has a large number of cirrhotic cases, the proportion of HCV is still relatively small. This major disparity in the etiology encountered in our center could underestimate the actual situation of HCV patients worldwide. Moreover, the remarkably high proportion of HCC patients in the cohorts, especially in HBV and ALD + HBV groups is worth explaining. This is mainly due to the fact that our center been a tertiary hospital receives mostly advanced liver disease patients. In addition, the high screening rate of HCC through CT scan, ultrasound and MRI make detection of early-stage HCC easy and frequent. Thus, the need of a multicentered large cohort prospective study.

## Supplementary Information

**Additional file 1**. **Supplementary table 1.** General characteristics of the studied groups. **Supplementary table 2.** Univariate and multivariate logistic regression evaluating factors associated with HCC in ALD, HCV and ALD+HCV patients. **Supplementary table 3.** Univariate and multivariate logistic regression evaluating factors associated with EGVB in ALD, HCV and ALD+HCV patients. **Supplementary table 4.** Relation between alcohol abstinence and risk of EGVB in ALD, ALD+HBV and ALD+HCV. **Supplementary table 5.** Relation between alcohol abstinence and risk of HCC in ALD patients (detailed). **Supplementary table 6.** Relation between alcohol abstinence and risk of EGVB in ALD patients (detailed). **Supplementary table 7.** Relation between alcohol abstinence and risk of HCC in ALD+HBV patients (detailed). **Supplementary table 8.** Relation between alcohol abstinence and risk of EGVB in ALD+HBV patients (detailed). **Supplementary table 9.** Relation between alcohol abstinence and risk of HCC in ALD+HCVpatients (detailed). **Supplementary table 10.** Relation between alcohol abstinence and risk of EGVB in ALD+HCV patients (detailed). **Supplementary figure 1.** Changing trend in the proportion of various liver cirrhosis etiology over the years. **Supplementary figure 2.** Age-sex distribution of the studied population. **Supplementary figure 3.** Proportion of HCC (fig A) and EGVB (fig B) in ALD, ALD+HBV and ALD+HCV patients according to the duration of alcohol abstinence.

## Data Availability

The datasets used during the current study are available from the corresponding author on reasonable request.

## Abbreviations

ALD: Alcohol-induced liver disease
HBV: Hepatitis B virus
HCV: Hepatitis C virus
ALD + HBV: Coexisting alcohol-induced liver disease and hepatitis B virus
ALD + HCV: Coexisting alcohol-induced liver disease and hepatitis C virus
EGVB: Esophageal gastric variceal bleeding
HCC: Hepatocellular carcinoma
MELD: Model for end-stage liver disease
GAHS: Glasgow Alcoholic Hepatitis Score
MDF: Maddrey’s discriminant function
HE: Hepatic encephalopathy
HRS: Hepatorenal syndrome
AVT: Antiviral treatment
