# Supplementary material for: Effect of alcohol on clinical complications of hepatitis virus-induced liver cirrhosis: a consecutive ten-year study
Source: BMC Gastroenterol. 2022 Mar 19;22:130. doi: 10.1186/s12876-022-02198-w (PMC8934474; doi:10.1186/s12876-022-02198-w)
Supplement: Supplementary file 1 — Additional file 1. Supplementary table 1. General characteristics of the studied groups. Supplementary table 2. Univariate and multivariate logistic regression evaluating factors associated with HCC in ALD, HCV and ALD+HCV patients. Supplementary table 3. Univariate and multivariate logistic regression evaluating factors associated with EGVB in ALD, HCV and ALD+HCV patients. Supplementary table 4. Relation between alcohol abstinence and risk of EGVB in ALD, ALD+HBV and ALD+HCV. Supplementary table 5. Relation between alcohol abstinence and risk of HCC in ALD patients (detailed). Supplementary table 6. Relation between alcohol abstinence and risk of EGVB in ALD patients (detailed). Supplementary table 7. Relation between alcohol abstinence and risk of HCC in ALD+HBV patients (detailed). Supplementary table 8. Relation between alcohol abstinence and risk of EGVB in ALD+HBV patients (detailed). Supplementary table 9. Relation between alcohol abstinence and risk of HCC in ALD+HCVpatients (detailed). Supplementary table 10. Relation between alcohol abstinence and risk of EGVB in ALD+HCV patients (detailed). Supplementary figure 1. Changing trend in the proportion of various liver cirrhosis etiology over the years. Supplementary figure 2. Age-sex distribution of the studied population. Supplementary figure 3. Proportion of HCC (fig A) and EGVB (fig B) in ALD, ALD+HBV and ALD+HCV patients according to the duration of alcohol abstinence. [file 12876_2022_2198_MOESM1_ESM.docx]

**Supplementary information**

**Materials and methods (detailed)**

**Criteria for diagnosis of each type of cirrhosis and its complications**

The diagnosis of liver cirrhosis was made through liver biopsy or imagery tests such as ultrasound, computerized tomography (CT) scan and magnetic resonance imaging (MRI), or through both biopsy and imaging.

ALD was diagnosed as documentation of regular alcohol consumption of more than 20 g/d in females and more than 40 g/d in males for at least five years, together with the presence of clinical and/or biological abnormalities suggestive of liver injury, after ruling out other etiologies of liver diseases [1].

Patients were considered as HBV patients when they have documented presence of HBV-DNA and/ or hepatitis B surface antigen for ≥ 6 months, after excluding other causes of liver damage.

HCV was diagnosed as presence of HCV-RNA and/ or positive anti-HCV antibodies after excluding other causes of liver damage.

ALD+HBV and ALD+HCV were respectively defined as cirrhotic HBV patient and cirrhotic HCV patient with excessive alcohol consumption (≥40g/d of ethanol in male, ≥20g/d of ethanol in female or ≥80g/week) for more than 5 years. The history of alcohol consumption of each patient, including duration of alcohol consumption, amount of daily consumption, duration of alcohol abstinence was obtained from reviewing the hospital electronic database of medical records. Patients with unclear history of alcohol intake were removed from the study.

Infection was considered in patients presenting evidence of infections commonly related to liver cirrhosis such as spontaneous bacterial peritonitis, urinary tract infection, pneumonia, bacteremia, soft tissue infection, etc. Ascites, HCC, esophageal gastric varices, EGVB and hepatic encephalopathy were registered according to the medical records, including laboratory results and imaging.

The diagnosis of diabetes was established by two fasting blood glucose determinations greater than 7.0 mmol/L.

Cirrhotic patients with one or more of the following chief complaints at admission were considered presenting liver cirrhosis decompensation complaints: GI bleeding symptoms such as melena and hematemesis, jaundice, ascites symptoms such as remarkable abdominal distention, or edema, central nervous system symptoms such as irritability, confusion, coordination or balance problem, mood or personality change, etc. Patients without any specific symptoms who were admitted due to abnormal laboratory findings such as abnormal liver function, abnormal coagulation function, elevated AFP, or liver mass on imaging were classified as non-decompensation complaints; so were those with non-related liver cirrhosis symptoms such as cardiovascular symptoms, respiratory symptoms, etc.

The proportion of EGV was calculated by dividing the number of patients reported with EGV by the number of patients who underwent upper GI endoscopy or other imaging examination capable of detecting EGV, such as CT or MR.

The proportion of EGVB was calculated by dividing number of patients reported with EGVB by the number of confirmed EGV patients.

MELD score was calculated as follow: 3.78×ln[serum bilirubin (mg/dL)] + 11.2×ln[INR] + 9.57×ln[serum creatinine (mg/dL)] + 6.43. A score greater or equal to 18 is associated with poor prognosis [2].

GAHS was calculated according to: patient’s age (< 50years old scores 1, ≥50year old scores 2), serum level of WBC (<15*10^9^/l scores 1, ≥15*10^9^/l scores 2), urea (<5 mmol/l scores 1, ≥5 mmol/l scores 2), INR (<1.5 scores 1, 1.5 to 2.0 scores 2, >2.0 scores 3), serum bilirubin (<125umol/l score 1, 125 to 250umol/l scores 2, >250umol/l score 3). A score greater or equal to 9 is associated with poor prognosis [3].

MDF was calculated as follow: 4.6*(prothrombin time – control time)+serum bilirubin [4]. A value greater or equal to 32 implies poor outcome [5].

Child-Pugh score was calculated according to: serum total bilirubin, serum albumin, INR, severity of ascites, severity of hepatic encephalopathy [6].

MCV>100, ALT>40, AST>40, GGT>60 was used to group patients into categorical variables. These cut-off values were chosen according to our hospital laboratory department cut-off values.

**Patients’ stratification and statistical analysis (detailed)**

We first compared the severity of liver function impairment between the groups using laboratory data such as ALT, AST, Child classification, MELD score, GAHS (Glasgow alcoholic hepatitis score) and MDS (Maddrey’s discriminant function Scale). The proportion of different liver cirrhosis complications such as HCC, infections, ascites, EGV, EGVB, hepatic encephalopathy (HE) and hepatorenal syndrome (HRS), was also evaluated among the groups. Multivariate logistic regressions were conducted to evaluate the association between different influencing factors such as etiology and the risk of HCC and EGVB. We then assessed the difference in the proportion of HCC and EGVB between males and females in each group. Next, patients of the ALD, ALD+HBV and ALD+HCV groups were stratified into four subgroups according to the duration of their abstinence from alcohol: no abstinence (subgroup 1), abstinent for less than five years (subgroup 2), abstinent for five to ten years (subgroup 3) and abstinent more than ten years (subgroup 4). Abstinence from alcohol was defined as restraint from any alcohol beverage for more than six months. Patients with unclear history of alcohol abstinence were excluded from this part of the study. The relation between alcohol abstinence and risk of HCC and EGVB was assessed in different groups through linear-by-linear association test and logistic regression analysis. Finally, to evaluate how antiviral treatment (AVT) affects the frequency of HCC and EGVB in the studied population, we divided HBV and HCV patients into two subgroups (patients with AVT, and those without AVT). The ALD+HBV patients and ALD+HCV patients were divided into 4 subgroups (patients without AVT and no alcohol abstinence, patients without AVT with alcohol abstinence, patients with AVT without alcohol abstinent and patients with AVT and alcohol abstinence). **Patients were classified as AVT patients when they met the following criteria: 1) HBV or ALD+HBV patients who had continuously received a conventional antiviral treatment with nucleos(t)ides analogues or peginterferon alfa for at least 12 months prior to admission and have a HBV-DNA level below 200 IU/mL. 2) HCV or ALD+HCV patients who had received conventional antiviral treatment with either peginterferon alfa or ribavirin, along with the addition of hepatitis C protease inhibitors, or patients who received the novel interferon-free regimens, and had attained sustained virological response (undetectable HCV RNA at admission). Patients were classified in the “no AVT” subgroup, when it is clearly mentioned in their admission chart that they were treatment naïve for HBV or HCV at admission. Non-compliant patients who discontinued treatment or had a HBV-DNA level greater than 200 IU/mL were also considered as no AVT patients. Patients with admission chart lacking information about AVT treatment were excluded from this part of the study. “Abstinent + AVT” was defined as patients with AVT who were abstinent from any alcohol beverage for at least 6 months prior to admission.** Covariates included in the multivariate logistic regressions were identified through univariate analysis. Statistical data were expressed as the median and 25^th^, 75^th^ percentiles or as percentage as appropriate. Kruskal-Wallis H test along with Bonferroni correction was used to compare quantitative variables between groups. Chi square (*X^2^*) test was used to compare categorical variables. All statistical tests were two-sided; *P* values below 0.05 were considered statistically significant. All data were analyzed using SPSS 25.0 (Armonk, NY: IBM corporation).

**References:**

1. Thursz M, Gual A, Lackner C, Mathurin P, Moreno C, Spahr L, et al. EASL Clinical Practice Guidelines: Management of alcohol-related liver disease. J Hepatol [Internet]. 2018;69(1):154–81. Available from: https://doi.org/10.1016/j.jhep.2018.03.018

2. Kamath PS, Wiesner RH, Malinchoc M, Kremers W, Therneau TM, Kosberg CL, et al. A model to predict survival in patients with end-stage liver disease. Hepatology. 2001;

3. Forrest EH, Evans CDJ, Stewart S, Phillips M, Oo YH, McAvoy NC, et al. Analysis of factors predictive of mortality in alcoholic hepatitis and derivation and validation of the Glasgow alcoholic hepatitis score. Gut. 2005;

4. Carithers RL, Herlong F, Diehl AM, Shaw EW, Combes B, Fallon HJ, et al. Methylprednisolone therapy in patients with severe alcoholic hepatitis. A randomized multicenter trial. Ann Intern Med. 1989;

5. Gholam PM. Prognosis and Prognostic Scoring Models for Alcoholic Liver Disease and Acute Alcoholic Hepatitis. Clinics in Liver Disease. 2016.

6. Pugh RNH, Murray‐Lyon IM, Dawson JL, Pietroni MC, Williams R. Transection of the oesophagus for bleeding oesophageal varices. Br J Surg. 1973;

**Supplementary tables and figures**

**Supplementary table 1. General characteristics of the studied groups**

| **Parameters** | **ALD**  **n=1652** | **HBV**  **n=18079** | **HCV**  **n=682** | **ALD+HBV**  **n=1594** | **ALD+HCV**  **n=280** |
| --- | --- | --- | --- | --- | --- |
| **Sex: male** | 1611 (97.5%) | 14914 (82.5%) | 391 (57.3%) | 1570 (98.5%) | 258 (92.1%) |
| **Age (years): median (P_25,_ P_75_)** | 52 (46, 59) | 52 (44, 61) | 55 (47, 64) | 53 (46, 60) | 46 (41, 53) |
| **Decompensation** | 975 (59.0) | 7039 (38.9) | 221 (32.4) | 888 (55.7) | 152 (54.3) |
| **Main chief complaint** |  |  |  |  |  |
| GI bleeding | 318 (19.2) | 1990 (11.0) | 87 (12.8) | 306 (19.2) | 66 (23.6) |
| Jaundice | 292 (17.7) | 2368 (13.1) | 20 (2.9) | 285 (17.9) | 38 (13.6) |
| ascites | 384 (23.2) | 2396 (13.2) | 110 (16.1) | 327 (20.5) | 56 (20.0) |
| CNS symptoms | 127 (7.7) | 947 (5.2) | 20 (2.9) | 104 (6.5) | 14 (5.0) |
| Abn routine check-up | 327 (19.8) | 2872 (15.9) | 234 (34.3) | 410 (25.7) | 70 (25.0) |
| Others | 353 (21.4) | 5801 (32.1) | 237 (34.7) | 301 (18.9) | 60 (21.4) |
| **alcoholism** |  |  |  |  |  |
| < 10 years | 309 (18.7) | - | - | 370 (23.2) | 92 (32.9) |
| 10-20 years | 631 (38.2) | - | - | 588 (36.9) | 114 (40.7) |
| > 20 years | 712 (43.1) | - | - | 636 (39.9) | 74 (26.4) |
| **Abstinence** |  |  |  |  |  |
| No abstinence | 1073 (64.9) | - | - | 1054 (66.1) | 204 (72.9) |
| < 5 years | 174 (10.5) | - | - | 261 (16.4) | 45 (16.1) |
| 5-10 years | 145 (8.8) | - | - | 145 (9.1) | 17 (6.1) |
| > 10 years | 260 (15.7) | - | - | 134 (8.4) | 14 (5.0) |
| **Use of AVT** | - | 863/10483  (8.2) | 431/682  (63.2) | 139/1006  (13.8) | 147/280  (52.5) |

NOTE: ALD, alcohol-induced liver disease; HBV, Hepatitis B virus; HCV, hepatitis C virus; ALD+HBV, co-existing ALD and HBV; ALD+HCV, co-existing ALD and HCV; “*” Patients with at least one chief complaint of decompensation (jaundice, GI bleeding, ascites or central nervous system signs and symptoms). “#” One single patient could present with multiple chief complaints; Abn routine check-up means abnormal routine check-up results such as transaminase, alfa-fetoprotein, abnormal liver mass on imaging, leading to admission. AVT, antiviral treatment.

**Supplementary table 2.** Univariate and multivariate logistic regression evaluating factors associated with HCC in ALD, HCV and ALD+HCV patients

| **Factors** | **Without HCC** | **With HCC** | **ULR** | **MLR** | |
| --- | --- | --- | --- | --- | --- |
|  | **n (%)** | **n (%)** | ***P* value** | **OR (95%CI)** | ***P* value** |
| Sex (male) | 1928 (87.4) | 332 (81.6) | *0.002* |  |  |
| Age (>50y) | 1222 (55.4) | 301 (74.0) | *<0.001* | 2.64 (1.88-3.71) | *<0.001* |
| **Etiologies** |  |  | *<0.001* |  |  |
| **HCV** | 540 (24.5) | 142 (34.9) |  | **1** |  |
| **ALD** | 1482 (67.1) | 170 (41.8) |  | **0.51 (0.35-0.74)** | ***<0.001*** |
| **ALD+ HCV** | 185 (8.4) | 96 (23.5) |  | **2.61 (1.63-4.18)** | ***<0.001*** |
| T2DM | 352 (16.9) | 72 (18.5) | *0.425* |  |  |
| Decomp. Sx. | 1195 (54.1) | 153 (37.6) | *<0.001* | 0.59 (0.42-0.81) | *0.001* |
| Infection | 686 (31.1) | 75 (18.4) | *<0.001* |  |  |
| Ascites | 1082 (49.0) | 206 (50.6) | *0.556* |  |  |
| HE | 149 (6.8) | 12 (2.9) | *0.003* |  |  |
| Thrombus | 113 (5.1) | 36 (8.8) | *0.003* | 5.04 (2.70-9.39) | *<0.001* |
| HRS | 56 (2.5) | 4 (1.0) | *0.054* |  |  |
| EGVB | 407 (39.4) | 64 (36.2) | *0.408* |  |  |
| Child classification |  |  | *<0001* |  |  |
| A | 675 (31.6) | 172 (43.3) |  |  |  |
| B | 965 (45.1) | 176 (44.3) |  |  |  |
| C | 498 (23.3) | 49 (12.3) |  |  |  |
| MCV >100 | 534 (24.3) | 64 (16.0) | *<0.001* | 0.62 (0.41-0.96) | *0.031* |
| ALT >40 | 958 (43.9) | 221 (55.3) | *<0.001* |  |  |
| AST >40 | 959 (94.1) | 217 (94.3) | *0.890* |  |  |
| GGT >60 | 787 (78.5) | 194 (84.3) | *0.049* | 2.06 (1.35-3.15) | *0.001* |

NOTE: ULR, univariate logistic regression; MLR, multivariate logistic regression; HCV, hepatitis C virus; ALD, alcohol-induced liver disease; ALD+HCV, co-existing ALD and HCV; HCC, Hepatocellular carcinoma; T2DM, type 2 diabetes mellitus; Decomp. Sx., decompensation symptoms and signs; HE, Hepatic Encephalopathy; EGVB, Esophageal gastric variceal bleeding, HRS: Hepatorenal Syndrome. MCV>100, ALT>40, AST>40, GGT>60 represent proportion of patients with mean corpuscular volume, alanine aminotransferase, aspartate aminotransferase, Gamma-glutamyl transferase greater than 100, 40, 40, and 60 respectively.

**Supplementary table 3.** Univariate and multivariate logistic regression evaluating factors associated with EGVB in ALD, HCV and ALD+HCV patients

| **Parameters** | **Without EGVB** | **With EGVB** | **ULR** | **MLR** | |
| --- | --- | --- | --- | --- | --- |
|  | **n (%)** | **n (%)** | ***P* value** | **OR (95%CI)** | ***P* value** |
| Sex (male) | 643 (87.2) | 433 (91.9) | *0.001* |  |  |
| Age (>50y) | 441 (59.8) | 254 (53.9) | *0.049* |  |  |
| **Etiologies** |  |  | *0.002* |  |  |
| **HCV** | 179 (24.3) | 87 (18.5) |  | **1** |  |
| **ALD** | 495 (67.1) | 318 (67.5) |  | **0.61 (0.33-1.40)** | ***0.121*** |
| **ALD+ HCV** | 64 (8.7) | 66 (14.0) |  | **2.91 (1.08-7.86)** | ***0.035*** |
| T2DM | 146 (19.8) | 53 (12.2) | *0.001* |  |  |
| Decomp. Sx. | 300 (40.7) | 471 (100) | *<0.001* |  |  |
| Infection | 163 (22.1) | 132 (28.0) | *0.020* |  |  |
| Ascites | 372 (50.4) | 257 (54.6) | *0.158* |  |  |
| HE | 42 (5.7) | 45 (9.6) | *0.011* |  |  |
| Thrombus | 51 (6.9) | 47 (10.0) | *0.057* |  |  |
| HCC | 113 (15.3) | 64 (13.6) | *0.408* |  |  |
| HRS | 9 (1.2) | 15 (3.2) | *0.017* |  |  |
| Child classification |  |  | *0.328* |  |  |
| A | 253 (35.0) | 138 (31.2) |  |  |  |
| B | 318 (44.0) | 199 (44.9) |  |  |  |
| C | 152 (21.0) | 106 (23.9) |  |  |  |
| MCV >100 | 175 (23.8) | 68 (14.5) | *<0.001* | 0.43 (0.24-0.78) | *0.006* |
| ALT >40 | 309 (42.0) | 178 (38.4) | *0.217* |  |  |
| AST >40 | 299 (96.5) | 177 (89.4) | *0.001* | 0.17 (0.04-0.78) | *0.023* |
| GGT >60 | 248 (80.8) | 152 (79.6) | *0.743* |  |  |

NOTE: ULR, univariate logistic regression; MLR, multivariate logistic regression; HCV, hepatitis C virus ; ALD, alcohol-induced liver disease; ALD+HCV, co-existing ALD and HCV; HCC, Hepatocellular carcinoma; T2DM, type 2 diabetes mellitus; Decomp. Sx., decompensation symptoms and signs; HE, Hepatic Encephalopathy; EGVB, Esophageal gastric variceal bleeding, HRS: Hepatorenal Syndrome. MCV>100, ALT>40, AST>40, GGT>60 represent proportion of patients with mean corpuscular volume, alanine aminotransferase, aspartate aminotransferase, Gamma-glutamyl transferase greater than 100, 40, 40, and 60 respectively.

**Supplementary table 4.** Relation between alcohol abstinence and risk of EGVB in ALD, ALD+HBV and ALD+HCV

|  | **Without EGVB** | **With EGVB** | **ULR** | **MLR** | |
| --- | --- | --- | --- | --- | --- |
|  | **n (%)** | **n (%)** | ***P* value** | **OR (95% CI)** | ***P* value** |
| **ALD** | | | | | |
|  |  |  | *0.001* |  |  |
| No abstinence | 364 (61.0) | 263 (83.8) |  | 1 |  |
| Abstinent <5 years | 83 (13.9) | 34 (10.8) |  | 0.58 (0.19-1.74) | *0.329* |
| Abstinent 5-10 years | 66 (11.1) | 14 (4.5) |  | 0.40 (0.13-1.30) | *0.129* |
| Abstinent >10 years | 84 (14.1) | 3 (1.0) |  | 0.06 (0.01-0.45) | *0.007* |
| **ALD+HBV** | | | | | |
|  |  |  | *<0.001* |  |  |
| No abstinence | 162 (40.4) | 219 (71.6) |  | 1 |  |
| Abstinent <5 years | 82 (20.4) | 50 (16.3) |  | 0.30 (0.17-0.54) | *<0.001* |
| Abstinent 5-10 years | 69 (17.2) | 17 (5.60) |  | 0.12 (0.06-0.26) | *<0.001* |
| Abstinent >10 years | 88 (21.9) | 20 (6.50) |  | 0.08 (0.04-0.16) | *<0.001* |
| **ALD+HCV** | | | | | |
|  |  |  | *<0.001* |  |  |
| No abstinence | 28 (44.4) | 54 (81.8) |  | 1 |  |
| Abstinent <5 years | 19 (30.2) | 8 (12.1) |  | 0.05 (0.01-0.26) | *<0.001* |
| Abstinent 5-10 years | 8 (12.7) | 3 (4.5) |  | 0.05 (0.01-0.43) | *0.006* |
| Abstinent >10 years | 8 (12.7) | 1 (1.5) |  | 0.03 (0.00-0.51) | *0.015* |

NOTE: ULR, univariate logistic regression; MLR, multivariate logistic regression; EGVB, Esophageal gastric variceal bleeding. The logistic regression analysis in ALD group was adjusted for alcoholism duration, presence of decompensation, diabetes, MCV, AST, presence of hepatic encephalopathy and HRS. In ALD+HBV group, the analysis was adjusted for sex, alcoholism duration, MCV, ALT, presence of decompensation, infection, HE, HCC and HRS. In ALD+HCV group, the analysis was adjusted for sex, alcoholism duration, presence of decompensation, child classification, thrombus and MCV. See supplementary tables 6, 8 and 10 for detailed results.

**Supplementary table 5.** Relation between alcohol abstinence and risk of HCC in ALD patients (detailed)

| **Factors** | **Without HCC** | **With HCC** | **ULR** | **MLR** | |
| --- | --- | --- | --- | --- | --- |
|  | **n (%)** | **n (%)** | ***P* value** | **OR (95%CI)** | ***P* value** |
| Sex (male) | 1444 (97.4) | 167 (98.2) | *0.526* |  |  |
| Age (>50y) | 840 (56.7) | 132 (77.6) | *<0.001* | 2.14 (1.36-3.34) | *<0.001* |
| **abstinence** |  |  | *0.001* |  |  |
| **No abstinence** | 942 (63.6) | 131 (77.1) |  | **1** |  |
| **< 5 years** | 156 (10.5) | 18 (10.6) |  | **0.71 (0.40-1.24)** | ***0.226*** |
| **5-10 years** | 136 (9.20) | 9 (5.30) |  | **0.37 (0.18-0.77)** | ***0.008*** |
| **> 10 years** | 248 (16.7) | 12 (7.1) |  | **0.27 (0.14-0.50)** | ***<0.001*** |
| Alcoholism |  |  | *<0.001* |  |  |
| < 10 years | 293 (19.8) | 16 (9.4) |  | 1 | *0.079* |
| 10-20 years | 575 (38.8) | 56 (32.9) |  | 1.82 (1.00-3.33) | *0.050* |
| > 20 years | 614 (41.4) | 98 (57.6) |  | 2.08 (1.13-3.83) | *0.018* |
| T2DM | 218 (15.6) | 27 (16.9) | *0.668* |  |  |
| Decomp. Sx. | 901 (60.8) | 74 (43.5) | *<0.001* | 0.58 (0.39-0.85) | *0.006* |
| Infection | 520 (35.1) | 33 (19.4) | *<0.001* | 0.55 (0.36-0.84) | *0.006* |
| Ascites | 791 (53.4) | 101 (59.4) | *0.135* |  |  |
| HE | 122 (8.20) | 5 (2.9) | *0.014* |  |  |
| Thrombus | 86 (5.80) | 16 (9.4) | *0.064* | 1.86 (1.02-3.40) | *0.042* |
| HRS | 46 (3.10) | 3 (1.8) | *0.330* |  |  |
| EGVB | 289 (39.7) | 29 (34.1) | *0.318* |  |  |
| Child classification |  |  | *0.002* |  |  |
| A | 358 (24.8) | 55 (34.0) |  |  |  |
| B | 677 (46.9) | 81 (50.0) |  |  |  |
| C | 407 (28.2) | 26 (16.0) |  |  |  |
| MCV >100 | 425 (28.8) | 28 (17.0) | *0.001* | 0.55 (0.35-0.86) | *0.008* |
| ALT >40 | 563 (38.2) | 80 (48.8) | *0.009* | 1.89 (1.33-2.67) | *<0.001* |
| AST >40 | 577 (94.3) | 82 (94.3) | *0.992* |  |  |
| GGT >60 | 533 (88.4) | 81 (93.1) | *0.189* |  |  |

NOTES: ULR, univariate logistic regression; MLR, multivariate logistic regression; ALD, alcohol-induced liver disease; HCC, Hepatocellular carcinoma; T2DM, type 2 diabetes mellitus; Decomp. Sx., decompensation symptoms and signs; HE, Hepatic Encephalopathy; EGVB, Esophageal gastric variceal bleeding, HRS: Hepatorenal Syndrome; MCV>100, ALT>40, AST>40, GGT>60 represent proportion of patients with mean corpuscular volume, alanine aminotransferase, aspartate aminotransferase, Gamma-glutamyl transferase greater than 100, 40, 40, and 60 respectively.

**Supplementary table 6.** Relation between alcohol abstinence and risk of EGVB in ALD patients (detailed)

| **Parameters** | **Without EGVB** | **With EGVB** | **ULR** | **MLR** | |
| --- | --- | --- | --- | --- | --- |
|  | **n (%)** | **n (%)** | ***P* value** | **OR (95%CI)** | ***P* value** |
| Sex (male) | 484 (97.8) | 311 (97.8) | *0.984* |  |  |
| Age (>50y) | 296 (59.8) | 181 (56.9) | *0.416* |  |  |
| **Abstinence** |  |  | *<0.001* |  |  |
| **No abstinence** | 292 (59.0) | 267 (84.0) |  | **1** |  |
| **< 5 years** | 76 (15.4) | 34 (10.7) |  | **0.58 (0.19-1.74)** | ***0.329*** |
| **5-10 years** | 62 (12.5) | 14 (4.40) |  | **0.40 (0.13-1.30)** | ***0.129*** |
| **> 10 years** | 65 (13.1) | 3 (0.90) |  | **0.06 (0.01-0.45)** | ***0.007*** |
| Alcoholism |  |  | *0.184* |  |  |
| < 10 years | 94 (19.0) | 50 (15.7) |  |  |  |
| 10-20 years | 174 (35.2) | 131 (41.2) |  |  |  |
| > 20 years | 227 (45.9) | 137 (43.1) |  |  |  |
| T2DM | 92 (18.6) | 29 (10.1) | *0.002* |  |  |
| Decomp. Sx. | 240 (48.5) | 318 (100) | *<0.001* |  |  |
| Infection | 131 (26.5) | 99 (31.1) | *0.149* |  |  |
| Ascites | 270 (54.5) | 183 (57.5) | *0.400* |  |  |
| HE | 35 (7.1) | 34 (10.7) | *0.071* |  |  |
| Thrombus | 39 (7.90) | 35 (11.0) | *0.130* |  |  |
| HCC | 56 (11.3) | 29 (9.1) | *0.318* |  |  |
| HRS | 9 (1.8) | 13 (4.10) | *0.052* |  |  |
| Child classification |  |  | *0.789* |  |  |
| A | 137 (28.4) | 87 (28.7) |  |  |  |
| B | 216 (44.8) | 129 (42.6) |  |  |  |
| C | 129 (26.8) | 87 (28.7) |  |  |  |
| MCV >100 | 135 (27.4) | 51 (16.0) | *<0.001* | 0.38 (0.19-0.78) | *0.008* |
| ALT >40 | 182 (37.0) | 106 (33.5) | *0.318* |  |  |
| AST >40 | 175 (95.6) | 109 (87.2) | *0.007* |  |  |
| GGT >60. | 162 (88.5) | 101 (84.9) | *0.355* |  |  |

NOTES: ULR, univariate logistic regression; MLR, multivariate logistic regression; ALD, alcohol-induced liver disease; HCC, Hepatocellular carcinoma; T2DM, type 2 diabetes mellitus; Decomp. Sx., decompensation symptoms and signs; HE, Hepatic Encephalopathy; EGVB, Esophageal gastric variceal bleeding, HRS: Hepatorenal Syndrome; MCV>100, ALT>40, AST>40, GGT>60 represent proportion of patients with mean corpuscular volume, alanine aminotransferase, aspartate aminotransferase, Gamma-glutamyl transferase greater than 100, 40, 40, and 60 respectively.

Supplementary table 7. Relation between alcohol abstinence and risk of HCC in ALD+HBV patients (detailed)

| **Factors** | **Without HCC** | **With HCC** | **ULR** | **MLR** | |
| --- | --- | --- | --- | --- | --- |
|  | **n (%)** | **n (%)** | ***P* value** | **OR (95%CI)** | ***P* value** |
| Sex (male) | 747 (98.0) | 823 (98.9) | *0.146* |  |  |
| Age (>50y) | 399 (52.4) | 598 (71.9) | *<0.001* | 6.25 (2.81-13.9) | *<0.001* |
| **abstinence** |  |  | *<0.001* |  |  |
| **No abstinence** | 370 (48.6) | 684 (82.2) |  | **1** |  |
| **< 5 years** | 163 (21.4) | 98 (11.8) |  | **0.10 (0.04-0.24)** | ***<0.001*** |
| **5-10 years** | 111 (14.6) | 34 (4.10) |  | **0.01 (0.00-0.04)** | ***<0.001*** |
| **> 10 years** | 118 (15.5) | 16 (1.90) |  | **0.01 (0.00-0.03)** | ***<0.001*** |
| Alcoholism |  |  | *<0.001* |  |  |
| < 10 years | 202 (26.5) | 168 (20.2) |  | 1 |  |
| 10-20 years | 307 (40.3) | 281 (33.8) |  | 0.19 (0.55-2.61) | *0.657* |
| > 10 years | 253 (33.2) | 383 (46.0) |  | 0.81 (0.32-2.07) | *0.667* |
| T2DM | 124 (16.3) | 84 (10.1) | *<0.001* | 0.30 (0.11-0.79) | *0.014* |
| Decomp. Sx. | 484 (63.5) | 404 (48.6) | *<0.001* | - | *-* |
| Infection | 293 (38.5) | 193 (23.2) | *<0.001* | 0.37 (0.18-0.75) | *0.006* |
| Ascites | 433 (56.8) | 446 (53.6) | *0.197* |  |  |
| HE | 79 (10.4) | 25 (3.0) | *<0.001* |  |  |
| Thrombus | 24 (3.1) | 56 (6.7) | *0.001* |  |  |
| HRS | 25 (3.3) | 10 (1.2) | *0.005* |  |  |
| EGVB | 110 (37.0) | 196 (47.8) | *0.004* | 0.42 (0.18-0.97) | *0.042* |
| Child classification |  |  | *<0.001* |  |  |
| A | 163 (21.7) | 326 (39.7) |  |  |  |
| B | 297 (39.5) | 347 (42.3) |  |  |  |
| C | 292 (38.8) | 148 (18.0) |  |  |  |
| MCV >100 | 190 (25.1) | 116 (14.0) | *<0.001* | 0.35 (0.17-0.74) | *0.006* |
| ALT >40 | 458 (60.4) | 521 (62.9) | *0.306* |  |  |
| AST >40 | 462 (95.1) | 499 (94.2) | *0.522* |  |  |
| GGT >60 | 381 (79.0) | 485 (92.4) | *<0.001* | 2.58 (1.14-5.82) | *0.023* |

**Supplementary table 8.** Relation between alcohol abstinence and risk of EGVB in ALD+HBV patients (detailed)

| **Parameters** | **Without EGVB** | **With EGVB** | **ULR** | **MLR** | |
| --- | --- | --- | --- | --- | --- |
|  | **n (%)** | **n (%)** | ***P* value** | **OR (95%CI)** | ***P* value** |
| Sex (male) | 399 (99.5) | 290 (94.8) | *<0.001* | 0.11 (0.02-0.61) | *0.012* |
| Age (>50y) | 260 (64.8) | 194 (63.4) | *0.692* |  |  |
| **Abstinence** |  |  | *<0.001* |  |  |
| **No abstinence** | 162 (40.4) | 219 (71.6) |  | **1** |  |
| **< 5 years** | 82 (20.4) | 50 (16.3) |  | **0.30 (0.17-0.54)** | ***<0.001*** |
| **5-10 years** | 69 (17.2) | 17 (5.6) |  | **0.12 (0.06-0.26)** | ***<0.001*** |
| **> 10 years** | 88 (21.9) | 20 (6.5) |  | **0.08 (0.04-0.16)** | ***<0.001*** |
| Alcoholism |  |  | *0.120* |  |  |
| < 10 years | 108 (26.9) | 62 (20.3) |  | 1 |  |
| 10-20 years | 136 (33.9) | 112 (36.6) |  | 2.04 (1.15-3.62) | *0.015* |
| > 20 years | 157 (39.2) | 132 (43.1) |  | 1.39 (0.80-2.42) | *0.244* |
| T2DM | 47 (11.7) | 28 (9.2) | *0.271* |  |  |
| Decomp. Sx. | 172 (42.9) | 306 (100) | *<0.001* |  |  |
| Infection | 81 (20.2) | 95 (31.0) | *0.001* |  |  |
| Ascites | 231 (57.6) | 177 (57.8) | *0.950* |  |  |
| HE | 15 (3.7) | 29 (9.5) | *0.002* |  |  |
| Thrombus | 23 (5.7) | 20 (6.5) | *0.659* |  |  |
| HCC | 214 (53.4) | 196 (64.1) | *0.004* |  |  |
| HRS | 3 (0.70) | 11 (3.6) | *0.007* |  |  |
| Child classification |  |  | *0.338* |  |  |
| A | 119 (30.0) | 78 (26.1) |  |  |  |
| B | 180 (45.3) | 134 (44.8) |  |  |  |
| C | 98 (24.7) | 87 (29.1) |  |  |  |
| MCV >100 | 76 (19.0) | 86 (28.4) | *0.003* |  |  |
| ALT >40 | 238 (59.5) | 150 (49.5) | *0.008* | 0.38 (0.24-0.61) | *<0.001* |
| AST >40 | 227 (95.4) | 153 (96.8) | *0.471* |  |  |
| GGT >60. | 195 (82.6) | 129 (83.8) | *0.769* |  |  |

**Supplementary table 9.** Relation between alcohol abstinence and risk of HCC in ALD+HCV patients (detailed)

| **Factors** | **Without HCC** | **With HCC** | **ULR** | **MLR** | |
| --- | --- | --- | --- | --- | --- |
|  | **n (%)** | **n (%)** | ***P* value** | **OR (95%CI)** | ***P* value** |
| Sex (male) | 180 (97.3) | 78 (82.1) | *<0.001* | 0.19 (0.05-0.69) | *0.011* |
| Age (>50y) | 40 (21.6) | 56 (58.9) | *<0.001* | 2.99 (1.43-6.25) | *0.004* |
| **abstinence** |  |  | *<0.001* |  |  |
| **No abstinence** | 119 (64.3) | 85 (89.5) |  | **1** |  |
| **< 5 years** | 38 (20.5) | 7 (7.4) |  | **0.26 (0.10-0.68)** | ***0.006*** |
| **5-10 years** | 15 (8.1) | 2 (2.1) |  | **0.17 (0.03-0.84)** | ***0.030*** |
| **> 10 years** | 13 (7.0) | 1 (1.1) |  | **0.10 (0.01-0.82)** | ***0.032*** |
| Alcoholism |  |  | *<0.001* |  |  |
| < 10 years | 81 (43.8) | 11 (11.6) |  | 1 |  |
| 10-20 years | 74 (40.0) | 40 (42.1) |  | 3.05 (1.37-6.81) | *0.006* |
| > 20 years | 30 (16.2) | 44 (46.3) |  | 3.78 (1.43-10.02) | *0.007* |
| T2DM | 30 (16.2) | 15 (15.8) | *0.927* |  |  |
| Decomp. Sx. | 108 (58.4) | 44 (46.3) | *0.055* |  |  |
| Infection | 59 (31.9) | 20 (21.1) | *0.056* |  |  |
| Ascites | 89 (48.1) | 55 (57.9) | *0.121* |  |  |
| HE | 10 (5.40) | 4 (4.2) | *0.644* |  |  |
| Thrombus | 3 (1.6) | 9 (9.5) | *0.002* |  |  |
| HRS | 3 (1.6) | 0 (0.00) | *0.212* |  |  |
| EGVB | 48 (55.8) | 18 (40.9) | *0.108* |  |  |
| Child classification |  |  | *0.775* |  |  |
| A | 54 (30.0) | 29 (30.9) |  |  |  |
| B | 91 (50.6) | 50 (53.2) |  |  |  |
| C | 35 (19.4) | 15 (16.0) |  |  |  |
| MCV >100 | 38 (21.0) | 29 (30.9) | *0.071* |  |  |
| ALT >40 | 99 (54.7) | 51 (54.3) | *0.944* |  |  |
| AST >40 | 100 (99.0) | 51 (98.1) | *0.630* |  |  |
| GGT >60 | 88 (88.0) | 43 (82.7) | *0.368* |  |  |

**Supplementary table 10.** Relation between alcohol abstinence and risk of EGVB in ALD+HCV patients (detailed)

| **Parameters** | **Without EGVB** | **With EGVB** | **ULR** | **MLR** | |
| --- | --- | --- | --- | --- | --- |
|  | **n (%)** | **n (%)** | ***P* value** | **OR (95%CI)** | ***P* value** |
| Sex (male) | 57 (89.1) | 64 (97.0) | *0.076* |  |  |
| Age (>50y) | 24 (37.5) | 23 (34.8) | *0.753* |  |  |
| **Abstinence** |  |  | *<0.001* |  |  |
| **No abstinence** | 28 (43.8) | 54 (81.8) |  | **1** |  |
| **< 5 years** | 20 (31.3) | 8 (12.1) |  | **0.05 (0.01-0.26)** | ***<0.001*** |
| **5-10 years** | 8 (12.5) | 3 (4.5) |  | **0.05 (0.01-0.43)** | ***0.006*** |
| **> 10 years** | 8 (12.5) | 1 (1.5) |  | **0.03 (0.00-0.51)** | ***0.015*** |
| Alcoholism |  |  | *0.658* |  |  |
| < 10 years | 21 (32.8) | 23 (34.8) |  |  |  |
| 10-20 years | 28 (43.8) | 24 (36.4) |  |  |  |
| > 20 years | 15 (23.4) | 19 (28.8) |  |  |  |
| T2DM | 10 (15.6) | 13 (19.7) | *0.543* |  |  |
| Decomp. Sx. | 17 (26.6) | 66 (100) | *<0.001* |  |  |
| Infection | 12 (18.8) | 16 (24.2) | *0.446* |  |  |
| Ascites | 29 (45.3) | 36 (54.5) | *0.293* |  |  |
| HE | 3 (4.7) | 7 (10.6) | *0.205* |  |  |
| Thrombus | 3 (4.7) | 2 (3.0) | *0.623* |  |  |
| HCC | 26 (40.6) | 18 (27.3) | *0.108* |  |  |
| HRS | 0 (0.00) | 2 (3.0) | *0.160* |  |  |
| Child |  |  | *0.063* |  |  |
| A | 27 (42.2) | 14 (22.6) |  |  |  |
| B | 28 (43.8) | 36 (58.1) |  |  |  |
| C | 9 (14.1) | 12 (19.4) |  |  |  |
| MCV >100 | 18 (28.1) | 7 (11.1) | *0.016* | 0.17 (0.03-0.87) | *0.034* |
| ALT >40 | 31 (48.4) | 32 (50.8) | *0.791* |  |  |
| GGT >60. | 27 (90.0) | 29 (90.6) | *0.934* |  |  |

**
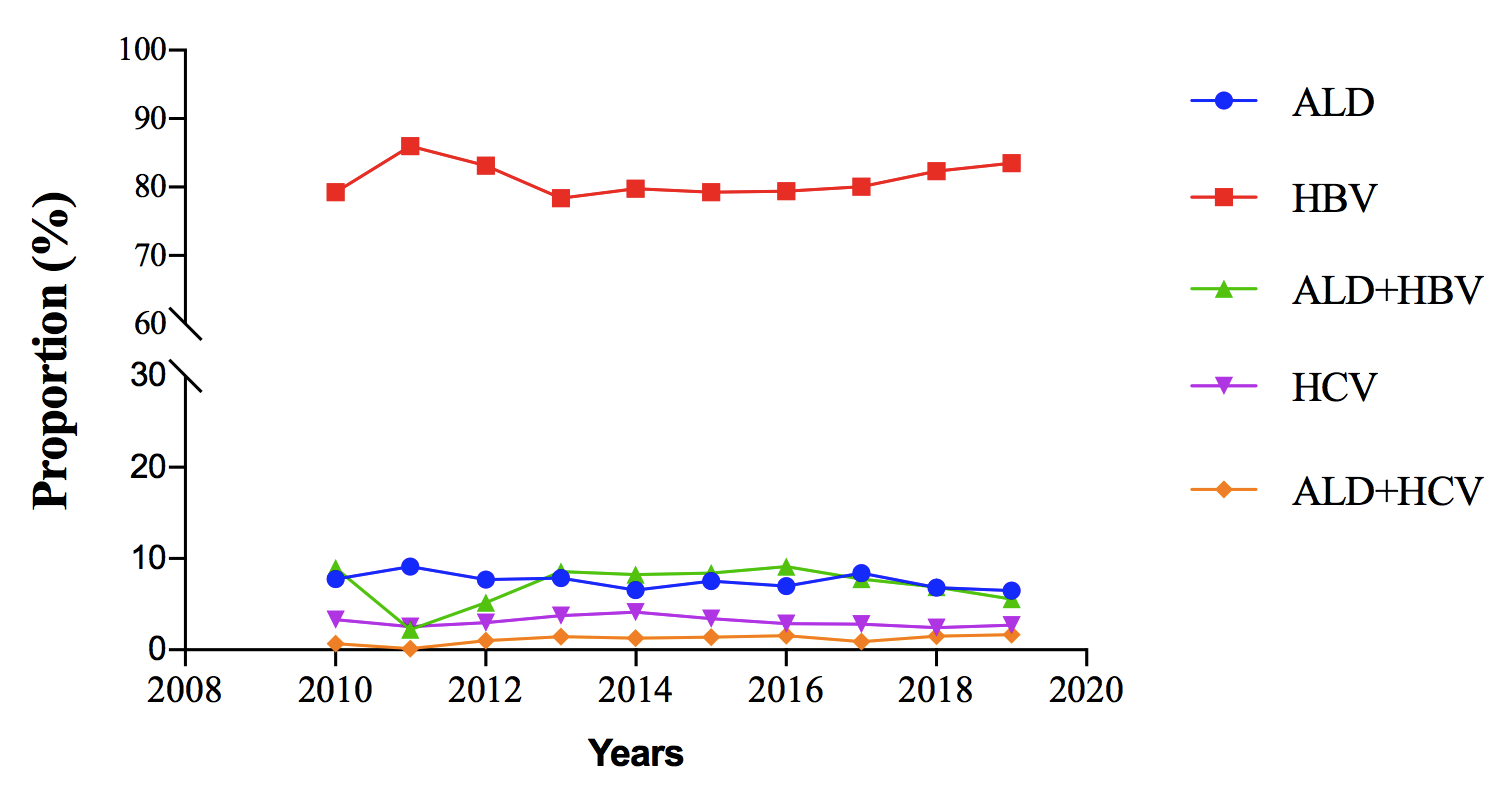
**

**Supplementary figure 1. Changing trend in the proportion of various liver cirrhosis etiology over the years.** ALD, alcohol-induced liver disease; HBV, hepatitis B virus; ALD+HBV, co-existing ALD and HBV; HCV, hepatitis C virus; ALD+HCV, co-existing ALD and HCV. HBV has been the major cause of cirrhosis, followed by ALD+HBV, ALD, HCV and ALD+HCV.


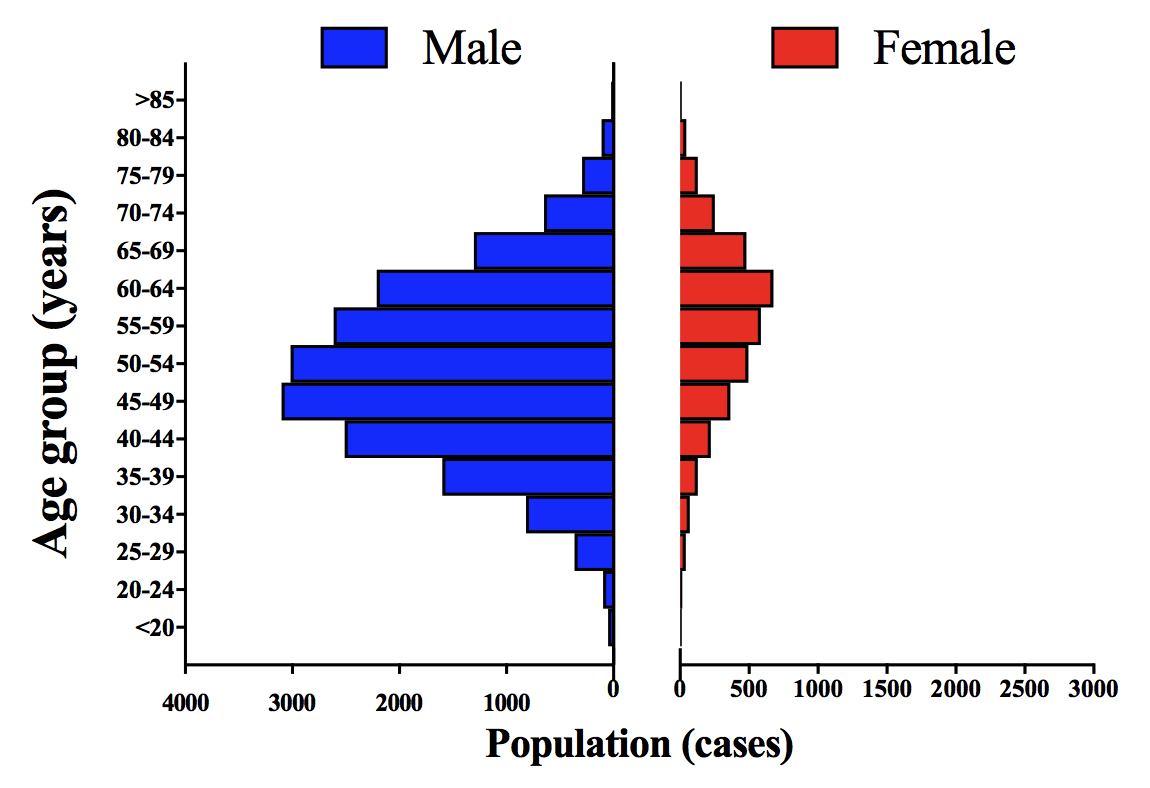


**Supplementary figure 2. Age-sex distribution of the studied population.** The average male patients were between 45-year-old to 55-year-old, median age 51 [43, 59] years, while the average female patients were between 55-year-old to 65-year-old median age 58 [50, 65] years, P<0.001.


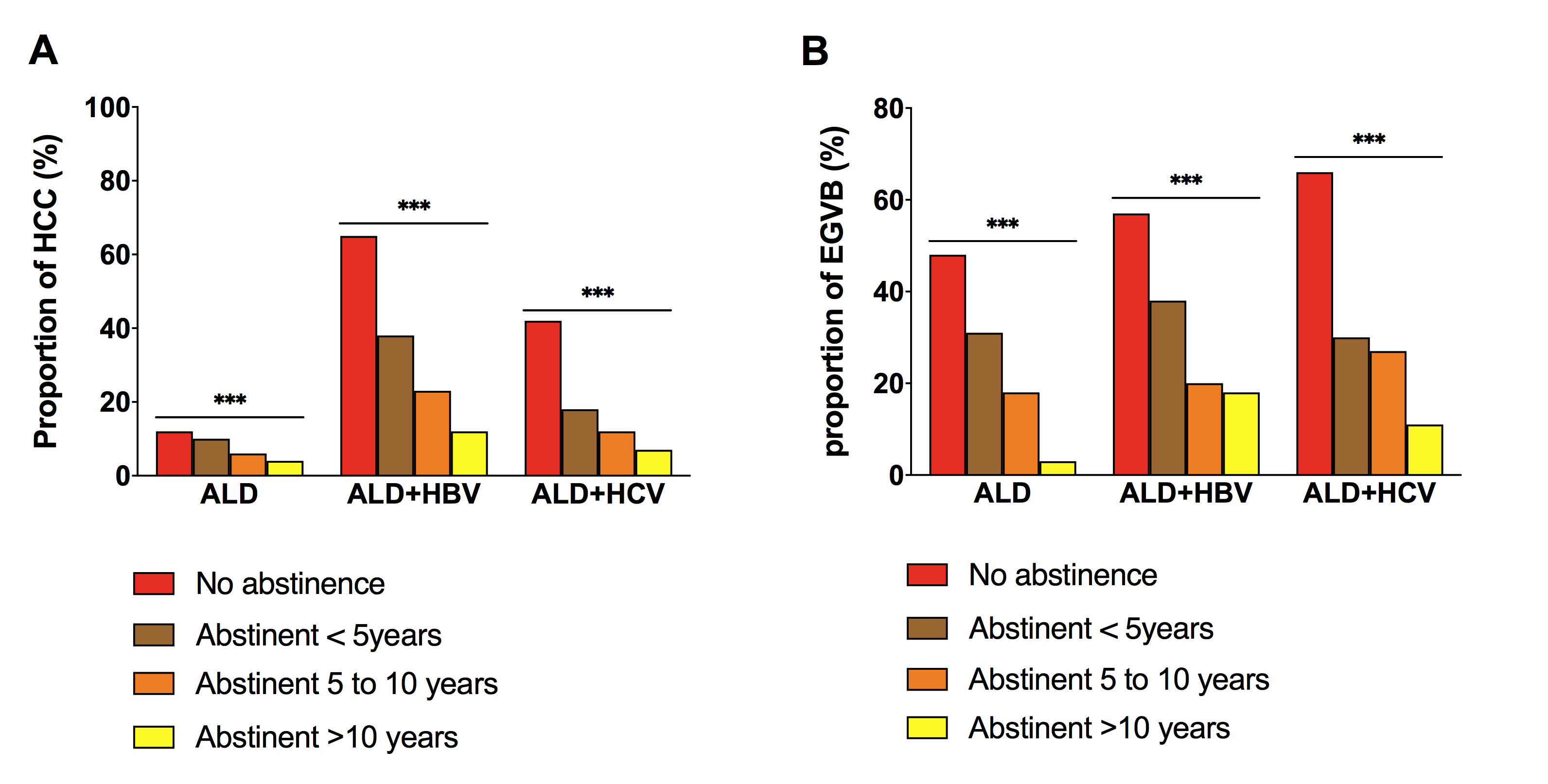


**Supplementary figure 3. Proportion of HCC (fig A) and EGVB (fig B) in ALD, ALD+HBV and ALD+HCV patients according to the duration of alcohol abstinence.** ALD, alcohol-induced liver disease; ALD+HBV, co-existing ALD and hepatitis B virus; ALD+HCV, co-existing ALD and hepatitis C virus; HCC, Hepatocellular carcinoma; EGVB, Esophageal gastric variceal bleeding. Linear-by-linear association test showed a significant decreased in the proportion of both HCC and EGVB with duration of alcohol abstinence (P<0.001).
